# Comprehensive Analysis of Serum Small Extracellular Vesicles-Derived Coding and Non-Coding RNAs from Retinoblastoma Patients for Identifying Regulatory Interactions

**DOI:** 10.3390/cancers14174179

**Published:** 2022-08-29

**Authors:** Radhika Manukonda, Vengala Rao Yenuganti, Nupur Nagar, Pankaj Singh Dholaniya, Shivani Malpotra, Jyothi Attem, Mamatha M. Reddy, Saumya Jakati, Dilip K Mishra, Pallu Reddanna, Krishna Mohan Poluri, Geeta K. Vemuganti, Swathi Kaliki

**Affiliations:** 1 The Operation Eyesight Universal Institute for Eye Cancer, L V Prasad Eye Institute, Hyderabad 500034, India; 2 Brien Holden Eye Research Center, L V Prasad Eye Institute, Hyderabad 500034, India; 3 Department of Animal Biology, School of Life Sciences, University of Hyderabad, Prof. C.R. Rao Road, Gachibowli, Hyderabad 500046, India or; 4 Department of Biosciences and Bioengineering, Centre for Nanotechnology, Indian Institute of Technology Roorkee, Roorkee 247667, India; 5 Department of Biotechnology and Bioinformatics, School of Life Sciences, University of Hyderabad, Prof. C.R. Rao Road, Gachibowli, Hyderabad 500046, India; 6 School of Medical Sciences, Science Complex, University of Hyderabad, Prof. C.R. Rao Road, Gachibowli, Hyderabad 500046, India; 7 The Operation Eyesight Universal Institute for Eye Cancer, L V Prasad Eye Institute, Bhubaneswar 751024, India or; 8 Ophthalmic Pathology Laboratory, L V Prasad Eye Institute, Hyderabad 500034, India

**Keywords:** retinoblastoma, extracellular vesicles, exosomes, liquid biopsy, RNA seq, microRNA, long non-coding RNA

## Abstract

**Simple Summary:**

The diagnosis of retinoblastoma (RB) is usually made by clinical examination and imaging modalities. Routine tissue biopsy is not done due to the risk of extraocular spread. Blood-based RNA cargoes could be promising surrogate markers for RB diagnosis and prognostication. Our data indicated that the size, morphology, and zeta potential (ZP) of RB and non-RB serum extracellular vesicles (EVs) met standard exosome properties with similar concentrations. MALTA1, AFAP1-AS1, miR-145, and miR-101 were identified as hub non-coding RNAs that promote RB progression by targeting cyclins, cyclin-dependent kinases, c-MYC, EZH2, ZEB1, TP53, and BCL2. Along with these, the aberrantly expressed miRNAs, lncRNAs, and their target mRNAs of RB EVs were implicated in cell cycle, metabolism, and tumor-associated signaling pathways. The differential expression of EV RNAs in RB compared to controls may aid in the identification of possible serum prognostic biomarkers for RB.

**Abstract:**

The present study employed nanoparticle tracking analysis, transmission electron microscopy, immunoblotting, RNA sequencing, and quantitative real-time PCR validation to characterize serum-derived small extracellular vesicles (sEVs) from RB patients and age-matched controls. Bioinformatics methods were used to analyze functions, and regulatory interactions between coding and non-coding (nc) sEVs RNAs. The results revealed that the isolated sEVs are round-shaped with a size < 150 nm, 5.3 × 10^11^ ± 8.1 particles/mL, and zeta potential of 11.1 to −15.8 mV, and expressed exosome markers CD9, CD81, and TSG101. A total of 6514 differentially expressed (DE) mRNAs, 123 DE miRNAs, and 3634 DE lncRNAs were detected. Both miRNA-mRNA and lncRNA-miRNA-mRNA network analysis revealed that the cell cycle-specific genes including CDKNI1A, CCND1, c-MYC, and HIF1A are regulated by hub ncRNAs MALAT1, AFAP1-AS1, miR145, 101, and 16-5p. Protein-protein interaction network analysis showed that eye-related DE mRNAs are involved in rod cell differentiation, cone cell development, and retinol metabolism. In conclusion, our study provides a comprehensive overview of the RB sEV RNAs and regulatory interactions between them.

## 1. Introduction

Retinoblastoma (RB) is an aggressive intraocular malignancy of childhood initiated by biallelic inactivation of the RB1 gene, and a small subset (1–2%) develops with MYCN amplification in the presence of functional RB1 [1,2]. The overall survival of RB patients is above 95% for the developed world, as the disease is intraocular at the time of diagnosis, compared to 50% to 90% in developing countries where the majority of the patients present with advanced intraocular disease (Groups D & E) or with extraocular tumor extension [3]. Since a tissue biopsy is associated with the risk of tumor dissemination, diagnosis, and treatment decisions for RB are based on clinical, imaging, and histopathological features [4]. Hence, there is an unmet need for identifying the prognostic biomarkers in RB.

Emerging evidence suggests that non-invasive liquid biopsies, particularly circulating extracellular vesicles (EVs), offer a promising alternative for tumor biopsy and aid in tumor characterization and identification of diagnostic and prognostic biomarkers for various cancers [5,6,7]. EVs are believed to exhibit intercellular communication by transferring their internal cargo (DNA, mRNAs, miRNAs, long non-coding RNAs (lncRNAs), lipids, and proteins) to recipient cells. Based on the size and biogenesis, EVs are categorized into small-sized EVs (30–150 nm), medium-sized EVs (100–1000 nm), and large-sized EVs (>1000 nm) [8]. Exosomes are small EVs (sEVs) involved in various normal and pathological conditions including cancer [9]. sEV RNAs are of considerable diagnostic interest as naked RNAs are unstable in blood compared to vesicle-enclosed RNAs [10]. Moreover, sEV mRNAs, miRNAs, and lncRNAs are shown to regulate target gene expression, alter cell metabolism and drive tumorigenesis [11]. Therefore, non-invasive sEV-based liquid biopsy and RNA analysis may serve as an alternative to tumor biopsies to evaluate the mechanism of tumor progression and for finding disease-associated biomarkers [12]. 

In pursuit of identifying EV-based biomarkers, various clinical trials have been completed for prostate cancer (NCT02702856, NCT03031418, NCT03235687, NCT04720599), and a few are in progress for lung (NCT05058768), oropharyngeal (NCT02147418), and ovarian cancers (NCT03738319) [13,14,15,16]. However, studies on RB EVs are limited and are confined to RB cell line, and primary cell cultures established from enucleated eyes of RB patients [17,18,19,20,21]. sEVs derived from WERI RB1 cells were shown to promote tumor growth and mediate tumor deterioration in RB murine xenograft model via miR-92a, 20a, 129a, and 17, C-X-C chemokine receptor type 4, and thrombospondin-1 [17]. In addition, RB-derived small EVs can also promote the angiogenesis of human vesicle endothelial cells via miR-92a-3p [19]. Moreover, only the shortlisted sEV miRNAs from RB cell lines have been validated in RB tumors and corresponding serum sEVs [21]. Nonetheless, the comprehensive analysis of total RNA contents (coding and non-coding (ncRNAs)) and their interactions in RB serum sEVs has not yet been studied. Thus, the present study aimed to evaluate the serum sEVs in RB patients and age-matched controls and analyze their RNA content by RNA sequencing. Regulatory networks of miRNA-mRNA and lncRNA-miRNA-mRNA were built for understanding complex molecular interactions involved in RB pathogenesis.

## 2. Materials and Methods

### 2.1. Sample Collection

This study was conducted according to the guidelines of the Declaration of Helsinki [22], and carried out after obtaining approval from the institutional review board (IRB) (LEC-BHR-P-01-21-575) of L V Prasad Eye Institute (LVPEI). Informed consent was obtained from the parents or legal guardians of the children involved in the study. The diagnosis of retinoblastoma (RB) was established based on clinical findings by examination under anesthesia, B-scan ultrasonography, and orbital imaging. Tumors were classified (group A–E) based on International Classification of Retinoblastoma (ICRB) classification system [23]. Blood samples (2 to 3 mL) were collected from treatment-naive RB patients (*n* = 9) and from healthy age-matched controls (*n* = 5) with no known retinal pathology. 

The collected blood samples were centrifuged at 2000× *g* for 15 min to remove any cellular debris. The supernatants containing the cell-free serum samples were stored in aliquots at −80 °C. Fresh RB tumor tissues (*n* = 5) were collected following enucleation of the eye as part of the treatment protocol for advanced intraocular tumor. An area of maximum tumor volume based on orbital imaging was identified, a 5 mm sclero-choroidal incision was given with a blade, the tumor was identified, and 2–3 mm of fresh tumor was obtained and transferred to an aliquot containing 400 µL of RNA later. The entire procedure was performed in the operation theater immediately after enucleation, and under sterile conditions. The enucleated globe was then submitted for routine histopathological examination. The control retinas (*n* = 2) were acquired from human cadaveric eyeballs, which were collected within 6 h of death and preserved in a sterile moist chamber in Ramayamma International Eye Bank at LVPEI, Hyderabad. The globe was bisected with blade adjacent to the optic nerve, the retina was identified, carefully dissected, and 2 mm of it was transferred to an aliquot containing 400 µL of RNA later and stored at −80 °C until RNA isolation. The remaining retina was sectioned and stained to rule out any evidence of retinal disease. 

### 2.2. Small Extracellular Vesicles Isolation from Serum

sEVs were recovered from serum samples using the commercial kit Total Exosome Isolation™ from serum (Invitrogen by Thermo Fisher Scientific, Vilnius, Lithuania) as per manufacturer’s instructions. Briefly, 100 µL of reagent was added to 500 µL of serum sample, and the solution was incubated overnight at 4 °C. Then precipitated vesicles were recovered by centrifugation at 10,000× *g* for 10 min. The pellet was dissolved in 100 μL PBS, aliquoted and stored at −80 °C for further experiments. The detailed methodology for comprehensive analysis of serum sEVs is illustrated in Figure S1. From all the 9 RB patients, sEVs were isolated and characterized for physical properties, but for RNA sequencing R4, R5 and R6 aged 5, 2, and 4 were selected. All three of them were male unilateral RB cases (Table S2). Among the five controls, we randomly picked first three controls aged 5, 5 and 4.

### 2.3. Transmission Electron Microscopy 

The morphology of sEVs was examined by TEM according to the technique described by Ahmed et al. [24]. Briefly, 20 μL of EVs PBS solution (1:100 dilutions) drop was loaded onto carbon coated copper grids and permitted to stand overnight for air drying. The absorbed sEVs were negatively stained with 2% uranyl acetate for 10 min. Finally, the images of sEVs were captured under TEM (JEOL JEM-1400Flash, Road Peabody, MA, USA) at 80 kV after the grids were dried.

### 2.4. Nanoparticle Tracking Analysis by Zeta View

All the purified single freeze-thawed sEV fractions were analyzed for particle size, concentration, and zeta potential using a ZetaView device (Particle Metrix GmbH, Meerbusch, Germany) and its corresponding software (version 8.05.12 SP1) [25]. Each sEV sample (1 µL in triplicates) was diluted (1:4000) in PBS, and 2 µL of this solution was injected into the cell. The instrument measured each sample at 11 different positions throughout the cell, with two cycles of readings at each position. The detection threshold of the zetaView software was set to 5, and the maximum jump distance, and the minimum track segment length were both set to auto. After automated analysis of all 11 positions, the mean, median and mode (indicated as diameter) sizes, ZP, as well as the concentration of the sample were calculated by the inbuilt software. As described earlier [26], we selected the mode as the measurement for size in our analysis. Final concentration was calculated by multiplying the observed concentration with the dilution factor. The concentration of EVs present in each sample was expressed as (particles/mL).

### 2.5. Immunoblotting for Exosome Specific Proteins

To demonstrate the presence of exosomal protein markers in serum small EV samples, immunoblotting was performed. Total exosomal protein concentration was estimated by a commercially available BCA kit (Thermo Fisher Scientific. Waltham, MA, USA) as per manufacture instructions. Then denatured proteins were separated on 10% SDS PAGE and transferred onto a PVDF membrane using a Trans-Blot^®^ SD Semi-Dry Transfer Cell (Bio Rad, Hercules, CA, USA). Membranes were blocked with 5% nonfat milk TBST for 1 h at room temperature and then incubated with primary antibodies anti-CD9 (D8O1A, Cell Signaling Technology, Danvers, MA, USA), anti- CD81 (EPR4244, Abcam, Cambridge, MA, USA), and anti TSG101 (EPR7130(B)), Abcam, Cambridge, MA, USA), overnight at 4 °C. The membranes were washed three times with 1 × TBST for 10 min and incubated with an HRP-conjugated secondary antibody (7074, Cell Signaling Technology, Danvers, MA, USA) for 2 h at room temperature, and washed in TBST. Signals were visualized after incubation with enhanced chemiluminescence kit by ChemiDoc (Bio-Rad, Hercules, CA, USA).

### 2.6. RNA Isolation, Library Preparation, Sequencing, and Data Processing

Large RNA (>200 nt) and small RNA (<200 nt) fractions were extracted from pooled sEV preparations of RB patients (*n* = 3) and age-matched controls (*n* = 3) using Total Exosome RNA & Protein Isolation Kit (Invitrogen™) following the manufacturer’s protocol. One mL PBS was added to the enriched sEV pellet. To this, 2% denaturation solution was added and mixed thoroughly. The mixture was incubated for 5 min at 4 °C. Next, one volume of acid-phenol-chloroform solution was added, the samples mixed by vortexing for 30–60 S and then centrifuged for 5 min at 10,000× *g* at room temperature. The aqueous fraction was collected, and 1/3 volume of 100% ethanol was added. The lysate was then passed through the filter cartridge and centrifuged for 30 S. At this stage, large RNA and small RNA were collected: the flow through contained small RNA and filter contained large RNA. Then, the large RNA filter was centrifuged for an additional 1 min at 10,000× *g*, transferred into a fresh collection tube, and 50 μL of nuclease-free water was added to the center of the filter. The samples were centrifuged for 30 S at 10,000× *g*. The elute containing the large RNA was collected and stored at −80 °C.

For small RNA recovery, a 2/3rd volume of ethanol was added to the flow-through containing the small RNA and mixed thoroughly. Ethanol mixture containing the small RNA was dropped onto the second filter cartridge, centrifuged for 30 S, and the same step repeated twice. The flow-through was discarded. The small-RNA bound cartridge was washed with wash buffer I, II and III, centrifuged, and the flow-through discarded. A volume of 50 µL of nuclease free water was added to the small RNA-bound cartridge and centrifuged for 30 S to recover the RNA. The eluate, which contained small RNAs was stored at −80 °C. Quality and quantity of the sEV RNAs were analyzed by NanoDrop^TM^ 2000 (Thermo Scientific). The large RNA fraction was subjected to whole transcriptome analysis (WTA) and the small RNA portion to small RNA sequencing. 

Large RNA sequencing libraries were constructed using NEBNext^®^ Ultra II Directional RNA Library Prep Kit (New England Biolabs, MA, USA) for Illumina^®^ as per the manufacturer’s protocol. The cDNA was subjected to end repair, and Illumina specific adaptors were ligated to the samples. The adaptor ligated products were then uniquely barcoded followed by PCR amplification. The libraries were quantified using a Qubit 4.0 fluorometer (Thermo Fisher Scientific. Waltham, MA, USA) using a DNA HS assay kit (Thermofisher). Sequencing was performed on an Illumina NovaSeq 6000 (Illumina, San Diego, CA, USA) with sequencing chemistry 2 × 150 bp. Small RNA libraries were prepared using Small RNA-Seq Library Prep Kit (LEXOGEN, Vienna, Austria) as per the manufacturer’s instructions. Final libraries were quantified using a Qubit 4.0 fluorometer (Thermofisher #Q33238) using a DNA HS assay kit (Thermofisher #Q32851) following the manufacturer’s protocol. Distribution of the fragment lengths of the obtained libraries were determined using high-sensitivity D1000 screen tapes (Agilent # 5067-5582) on Tapestation 4150 (Agilent) and cDNA selection was carried out according to the miRNA size using AMPure XP Beads (BioLabs Inc., San Diego, CA, USA) according to the NEB. 

Sequencing was performed on the NovaSeq 6000 platform using a NovaSeq 6000 S4 reagent kit v1.5. Adapter trimming and quality-based filtering (Phred score > 30) was done using fastp (v0.20) [27]. The clean reads were then aligned to the hg38 reference genome using HISAT2 (v 2.1.0) accessed on 12 September 2021 [28]. Transcript abundance estimates were measured in FPKM. LncRNAs were identified using databases (RNACentral (https://rnacentral.org/ accessed on 12 February 2022), and Gencode (www.gencodegenes.org/ accessed on 15 January 2022). For known miRNA detections, the clean reads were aligned against the miRNA precursor/mature miRNA in miRBase20.0 (http://www.mirbase.org/ accessed on 12 February 2022). Small RNAs such as tRNA and piRNA were identified using PirBase (http://bigdata.ibp.ac.cn/piRBase/), ENA (https://www.ebi.ac.uk/ena/browser/home), Rfam (https://rfam.xfam.org/), GtRNAdb (http://gtrnadb.ucsc.edu/), GeneCards (https://www.genecards.org/). The unaligned reads were used for the identification of novel miRNAs using miRDeep2 (https://github.com/rajewsky-lab/mirdeep2). All the databases were accessed on 12 February 2022.

### 2.7. Differential Expression Analysis

Differential expression analysis of mRNAs, lncRNAs and miRNAs was performed using EdgeR package (v 3.28.1) that counted data using a negative binomial distribution, and implemented statistical methods proposed by Robinson and Smyth [29]. Individual gene expression was calculated as the mean expression of each gene averaged over all samples of each group and presented as the logarithm of counts per million reads. A *p*-value cutoff of 0.05 and log2foldchange of (+/−) 2 were used for identifying significantly differentially regulated transcripts. The Benjamini Hochberg procedure, proposed by Benjamini and Hochberg was used to control the false discovery rate (FDR) [30].

### 2.8. miRNA and lncRNA Target Analysis

Target genes for the known DE miRNAs were identified by experimentally validatedmiRNA–target interaction database miRTarBase (http://miRTarBase.cuhk.edu.cn/) accessed on 16 February 2022 [31]. Then, miRNA-target enrichment analysis, and statistical significance associated with each miRNA-target interaction were analyzed by MIENTURNET (MicroRNA ENrichment TURned NETwork) (http://userver.bio.uniroma1.it/apps/mienturnet/) [32]. LncRNA2Target v2.0 (http://123.59.132.21/lncrna2target) [33], and LncTarD (http://bio-bigdata.hrbmu.edu.cn/LncTarD) accessed on 16 February 2022 [34] tools were used for identifying experimentally validated lncRNA targets. Along with these, the LncBase database in RNACentral (https://rnacentral.org/) accessed on 16 February 2022 was used for experimentally verified and computationally predicted microRNA targets on lncRNAs [35].

### 2.9. Functional Enrichment Analysis

The ClusterProfiler package (http://bioconductor.org/packages/release/bioc/html/clusterProfile-r.html) [36] was used to identify and visualize the top GO terms and KEGG pathways enriched by DE mRNAs and DE lncRNAs. The target genes of miRNAs were selected for GO analysis using GOnet tool http://tools.dice-database.org/GOnet/ accessed on 16 February 2022) [37]. Gene set enrichment analysis (GSEA) was performed using gene pathways extracted from GO and KEGG. Values were derived for the set of genes by permuting the gene sets for ‘n’ number of times within the available datasets. Cohesive rankings and differences in pathways were assigned by the GSEA algorithm. Validation of rankings and differences was done by calculating the statistical significance of the normalized enrichment score using nominal *p*-value and FDR q-value. The cutoffs for *p*-value and FDR q-value were set to 0.05 and 0.25.

### 2.10. Construction of RNA Interaction Networks

The interaction network of DE miRNAs and their target genes identified in RB serum small EVs was generated by miRTargetLink 2.0 (https://www.ccb.uni-saarland.de/mirtargetlink2 accessed on 3 March 2022) with parameter setting to strong interaction and minimum 5 shared targets options [38]. The associated biological processes were extracted by GeneTrail3.0 (http://genetrail.bioinf.uni-sb.de accessed on 3 March 2022) [39]. The miRNA-target network analysis was carried out using the tool cytoscape with 10 selected miRNAs that target the gene of interest RB1. An LncRNA-miRNA-mRNA interaction network was constructed on the basis that 23 selected lncRNAs bind to their target genes (either to mRNAs or miRNAs or to both). The network was visualized using cytoscape software (v 3.8.2; accessed on 15 March 2022) [40]. In this network, each node represented a biological molecule, and the edges were defined as interactions between nodes [41]. LncRNAs, mRNAs, and miRNAs in the network were presented as green triangles, blue rectangles, and pink ovals, respectively. Hub RNAs was selected based on the topological features of the network such as betweenness centrality, closeness centrality network and degree layout calculated by a built-in NetworkAnalyzer tool in Cytoscape software [42]. The functionally enriched KEGG pathway and GO terms for the network were visualized using the ClueGO/CluePedia plugin from Cytoscape [43,44].

### 2.11. Construction of Protein-Protein Interaction Network

A total of 39 DE mRNAs (20 up and 19 down regulated coding mRNAs) related to ocular development were filtered from core DE mRNAs of RB sEVs. We used a string database (version: 11.5 available at: https://string-db.org/ accessed on 20 March 2022) for construction of the protein interaction (PPI) network [45]. The protein names for the selected genes were given as input data by selecting multiple proteins by names/identifiers and interactions pertaining to Homo sapiens. The topological and functional properties of the PPI network were analyzed by K-means clustering, an unsupervised learning algorithm [46]. The resulting clusters were separated manually for better visual representation and comprehension of the interaction network. The functional significance and statistical analysis of the network were investigated automatically by inbuilt String software against the statistical background of the whole genome.

### 2.12. Quantitative Reverse Transcriptase-Polymerase Chain Reaction

Among the total DE mRNAs identified in RB sEVs by RNA sequencing, the expressions of HIF1A, SYK, and PGK1 were analyzed for their expression in corresponding tumor tissues by RT-qPCR. RB tissues and retinas were thawed, and total RNA was recovered using TRIZIN reagent (GCC Biotech Pvt. Ltd., West Bengal, India). The samples were homogenized in trizin and incubated at room temperature for 5 min. Chloroform was added and the tubes were mixed vigorously for 15 S and incubated at room temperature for 2–3 min. Next, the samples were centrifuged at 12,000× *g* for 15 min at 4 °C. The aqueous phase was precipitated with isopropanol, followed by 75% ethanol washes. After the washes, the RNA pellet was air dried and dissolved in nuclease fee water. The isolated RNA was quantified using NanoDrop^TM^ 2000 (ThermoFisher Scientific, Waltham, MA, USA). cDNA was prepared from 1 µg of RNA using RevertAid First Strand cDNA Synthesis kit (Thermo Scientific) using oligo (dT)18 primer and random hexamer primer according to the manufacturer instructions. qPCR was performed on a 7900HT Fast RT PCR system (Applied Biosystems, MA, USA) using DyNAmo™ Flash SYBR Green qPCR Kit (ThermoFisher Scientific MA, USA) as per manufacturer’s instructions. The reaction was performed in 96-well transparent plates (Thermo Fisher Scientific) for real time in a final volume of 10 μL. For each gene, three technical replicates of each sample were analyzed along with negative controls and a 5-point relative standard curve and the non-template control. The following amplification conditions were used: 10 min at 95 °C, 40 cycles of 15 S at 95 °C (denaturation), and 1 min at 60 °C (annealing and extension). A dissociation protocol with incremental temperatures of 95 °C for 15 S plus 65 °C for 15 S was used to investigate the specificity of the qPCR reaction. The qPCR expression data for each reference gene were extracted in the form of crossing points. The data acquired was computed by SDS software v2.3 (Applied Biosystems, Waltham, MA, USA) and subjected to subsequent analysis. The specificity and integrity of the PCR product was confirmed by a single melt curve peak. Relative expression was normalized to βactin and calculated according to the 2^−ΔΔCt^ approach [47]. The primer sequences used for detecting the expression of HIF1A, SYK, PGK1, and β-actin are given in Table S1.

### 2.13. Statistical Analysis

Data are presented as the mean ± SEM and statistically significant differences were identified with Student’s *t* test as indicated in the figure legends. The difference in size, concentration and ZP of RB and non-RB were analyzed by the Welch *t*-test. Student’s *t* test was used to compare the normalized mRNA expression levels and two-sided *p* < 0.05 was considered to be statistically significant.

## 3. Results

The demographic and clinical features of RB and non-RB subjects are summarized in Table S2. All the nine RB patients had intraocular unilateral RB with two belonging to Group D and 7 to Group E based on ICRB classification. The mean (median; range) age of RB children and controls were 3.6 (4; 1 to 9 years) and 5.4 (5; 4 to 8 years) respectively. Of the nine RB children, six were male and three were female, and amongst the five controls, three were male and two were female.

### 3.1. Characterization of Serum-Derived Extracellular Vesicles

**Figure 1:**
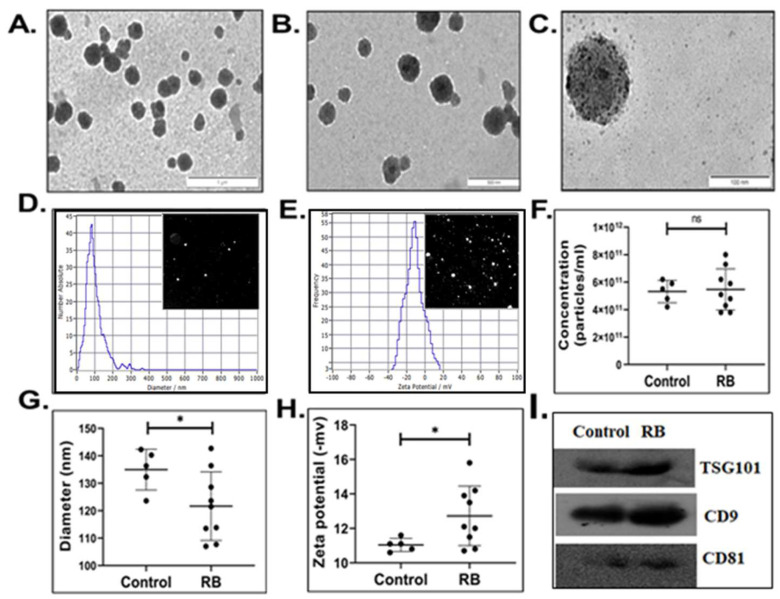
Representative transmission electronic images of small EVs (sEVs) recovered from Retinoblastoma (RB) RB serum samples. Images were captured at different scales (**A**) 1000 nm (**B**) 500 nm (**C**) 100 nm. (**D**) Size distribution profile and (**E**) Zeta potential of serum sEVs generated by ZetaView^®^ Nanoparticle Tracking Analyser (NTA). This captures the video data of sEVs moving under Brownian motion in PBS. The video data was then analysed using the NTA software (version 8.05.12 SP1). Dot plots representing (**F**) Concentration (particles/mL), (**G**) size distribution (nm), and (**H**) zeta potential (mV) of RB and non-RB serum EVs. Black circles indicates number of subjects included in each group, * Significance level: *p*-value < 0.05 calculated by Welch’s T-test, and ns stands for not significant. (**I**) Immunoblotting for exosome specific proteins.

The isolated single freeze-thaw cycled serum small EVs (sEVs) from RB and non-RB subjects were characterized by TEM, Zeta view, and immunoblotting. TEM results showed typical round-shaped membrane vesicles with a diameter range of 30–150 nm (Figure 1A–C). Over 95% of the recovered sEVs were smaller than 200 nm, most of them being in the size range of 30–150 nm characteristic of exosomes (Figure 1D). They had a net negative charge of <20 mV (Figure 1E). Average size (±SD), zeta potential (ZP) (±SD) and concentration (±SD) of sEVs per 1 mL for RB and non-RB groups were 135 ± 7.4 vs. 121.6 ± 12.5 (*p* = 0.03); −11.04 ± 0.4 mV vs. 12.72 ± 1.7 mV (*p* = 0.02), and 5.3 × 10^11^ ± 0.8 × 10^11^ vs. 5.47 × 10^11^ ± 1.5 × 10^11^ (*p* = 0.8), respectively (Figure 1F–H) and (Table S3). Immunoblotting results revealed the expression of exosome specific transmembrane proteins CD9 and CD81, and component of the ESCRT-I complex (TSG101) in serum sEVs (Figure 1I).

### 3.2. Analysis of Serum sEVs RNA Content by RNA Sequencing

**Figure 2:**
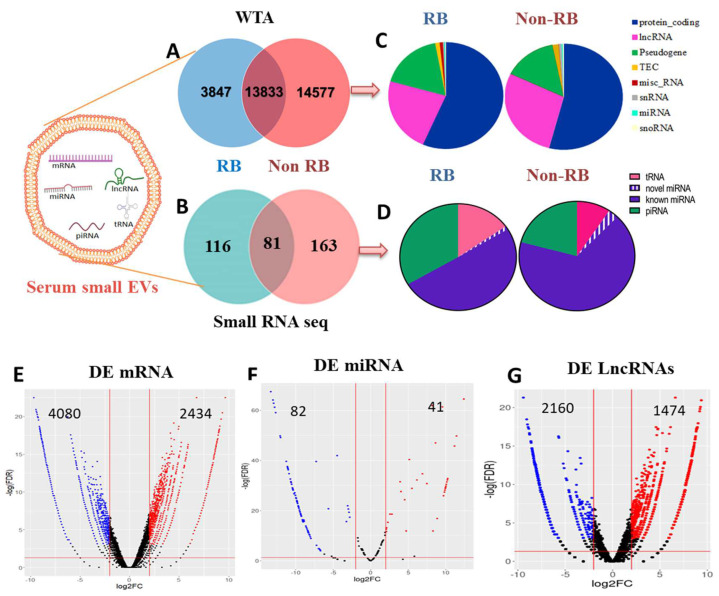
Total RNA profiles and distribution of RNA biotypes present in serum-derived small extracellular vesicles (sEVs) of retinoblastoma (RB) and age-matched controls (non-RB). (**A**,**B**) The Venn diagram depicts the fractions of unique and shared transcripts by RB and non-RB sEVs. (**C**,**D**) Pie charts show RNA categories of sEVs identified by WTA and small RNA sequencing. Volcano plot of differentially expressed (DE) (**E**) mRNAs, (**F**) microRNAs, and (**G**) long non-coding RNAs of retinoblastoma (RB) vs. non -RB serum-derived small EVs identified by EdgeR. Blue dots >= Down regulated genes; Red dots: upregulated genes; Black >= neural based on *p* < 0.05 and log2foldchange of (+/−) 2 criteria.

sEVs large and small RNA fractions recovered from RB and non-RB subjects were analyzed by WTA and small RNA sequencing. The sequencing details of both the analyses are given Table S4. WTA data revealed 3847 vs. 14,577 unique transcripts with 13,833 common transcripts (Figure 2A) and small RNA seq identified 116 vs. 163 unique transcripts with 81 common transcripts in RB and non-RB sEVs, respectively (Figure 2B). Large RNA fraction constituted mainly protein coding RNA (10,691 (57%) vs. 15,138 (54%)), lncRNA (4308 (23%) vs. 7883 (28%)), pseudogenes (3345 (18%) vs. 4119 (15%), and negligible fractions of miRNAs, snRNAs, and snoRNAs in RB vs. non-RB sEVs, respectively (Figure 2C). Small RNA fraction in RB vs. non-RB sEVs contained miRNAs (101 (51.3%) vs. 170 (69.7%)), piRNAs (66 (33.5%) vs. 51 (20.9%), and tRNAs (30 (15.2%) vs. 23 (9.4%)), respectively (Figure 2D). Of the 101 miRNAs detected in RB EVs, 99 were known and 2 were novel miRNAs, and of the 170 miRNAs detected in non-RB sEVs, 163 were known and 7 were novel miRNAs.

### 3.3. Identification of Differentially Expressed mRNAs, miRNAs and lncRNAs in RB sEVs

Based on the Log2FC (+/−) 2 and *p* < 0.05 criteria, 6514 DE mRNAs (2434 up and 4080 down), 115 known (35 up and 80 down), and 8 novel DE miRNAs (6 up and 2 down), and 3634 DE lncRNAs (1474 up and 2160 down) were identified from RB vs. non-RB sEVs. The DE of these RNAs was represented in individual volcano plots (Figure 2E–G). DE analysis of RNAs and their functional enrichment results were described below. 

### 3.4. Functional Enrichment Analysis of Differentially Expressed mRNAs and Protein-Protein Interaction-Network of Eye-Related Genes in RB Serum-Derived Small EVs

**Figure 3:**
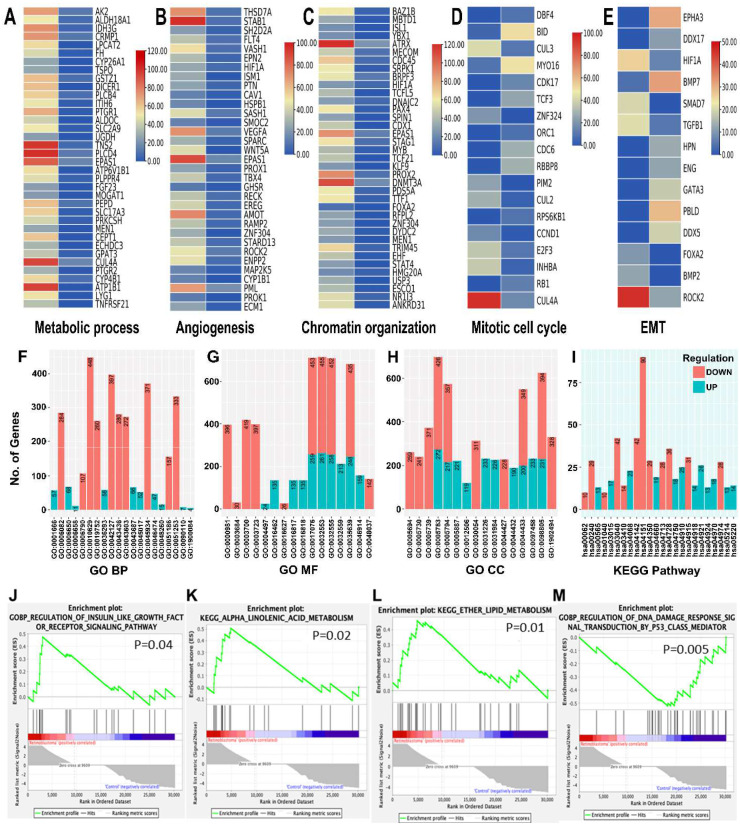
Heat maps showing differential expression (DE) of retinoblastoma (RB) serum small extracellular vesicles (sEVs) mRNAs associated with biological processes related to (**A**). Metabolic process, (**B**). Angiogenesis, (**C**). Chromatin organization, (**D**). Mitotic cell cycle, and (**E**). epithelial to mesenchymal transition (EMT). (**F**–**H**) Gene ontology (BP—Biological process, MF—Molecular function, CC—Cellular component) analysis, and (**I**). Kyoto Encyclopedia of Genes and Genomes (KEGG) enrichment results for DE mRNAs. (**J**–**L**) Selected Gene Set Enrichment Analysis plots showing gene sets enriched for RB sEVs, and (**M**) non-RB sEVs.

The top 20 up and down DE mRNAs in RB sEVs with their functions are listed in (Tables S5 and S6). Apart from these, DE mRNAs involved in mitotic cell-cycle regulation (RB1, CCND1, E2F3 and CUL3), metabolism (AK2, PLCD4, CUL4A and ATP1B1), chromatin organization (DMT3, PROX2 and ATRX), angiogenesis (STAB1, VEGFA, EPAS1, AMOT), and epithelial to mesenchymal transition (EMT) (EPHA3, SMAD7, ROCK2, and TGFB1) are represented as heat maps (Figure 3A–E). 

The enriched GO and KEGG terms for both up and down regulated DE mRNAs are represented in bar graphs (Figure 3F–I). The top enriched GO terms include negative regulation of gene expression (GO:0010629) and regulation of cell population proliferation (GO:0042127). Phosphatidylinositol 3′–kinase (PI3K)-Akt (hsa04151), transforming growth factor (TGF)-beta (hsa04350), Oxytocin (hsa04921), Insulin (hsa04910) and FoxO (hsa04068) signaling pathways, and phototransduction (hsa04744) are the enriched KEGG terms for RB sEVs DE mRNAs. GSEA results revealed that the top significant gene sets enriched for RB sEVs are associated with ether lipid metabolism (*p* = 0.01), alpha linolenic acid metabolism (*p* = 0.02), and regulation of insulin like growth factor receptor signaling pathway (*p* = 0.04) (Figure 3J–L). Regulation of DNA damage response signal transduction by p53 class mediator gene set is enriched for non-RB sEVs (*p* = 0.005) (Figure 3M). 

We also identified 39 DE mRNAs associated with eye development in RB sEVs (Table 1 and Table 2). A PPI network with these genes was examined for deciphering the functional role of these protein interactions in RB pathobiology. The PPI network of RB sEVs was divided into three significant clusters (Figure S2). Cluster 1 proteins were shown to be involved in 9-cis retinoic acid biosynthesis (*p* = 0.003) and retinol metabolic process (FDR = 3.58 × 10^−5^). Cluster 2 was enriched with retina development in camera-type eye (FDR = 1.20 × 10^−9^). The biological processes in cluster 3 were involved in phototransduction (FDR = 2.24 × 10^−6^), retinal rod cell differentiation (FDR = 0.02) and retinal cone cell development (FDR = 0.02). The uniprot annotated keywords enriched for the PPI network are KW-0844: Vision (FDR = 7.75× 10^−5^), KW-0716: Sensory transduction (FDR = 0.02) and KW-0238: DNA-binding (FDR = 0.02).

### 3.5. Differentially Expressed miRNA-Target Gene Analysis and Functional Enrichment

Computational analysis revealed that of 115 known DE miRNAs detected in RB sEVs, 42 up and 30 down regulated miRNAs have targets. The top dysregulated miRNAs belong to miR-17 (17-5p, 20a-5p, 106b-5p, 20b-5p), miR-15 (15b-5p, 15a-5p and 16-5p), and let-7 (98-5p) families contain highest number of targets (Tables S7 and S8). A heat map of the top 40 DE miRNAs in RB sEVs vs. non-RB is represented in Figure S3A. The miRNA-target enrichment results revealed that c-MYC, FGF2, SMAD3, JAK1, BCl2 and other cell cycle genes (CCND1, CDKN1A, CCND2, WEE1, E2F3 and PTEN are targeted by maximum number of both up and down regulated miRNAs (Figure S3B,C). RB1, the common dysregulated gene in RB was found to be regulated by 4 up (miR-17-5p, 20a-5p, 132-3p, 215-5p; FDR = 0.03) and 6 down miRNAs (23b-3p, 106b-5p, 192-5p, 130b-3p, 221-3p, 20b-5p; FDR = 0.04) (Figure S3D). The binding sites for these miRNAs on 3′UTR of RB1 are illustrated in Figure S3E. MYCN, the most common amplified gene in RB was shown to be targeted by 4 upregulated miRNAs (101-3p, 29a-3p, 19b-3p and 19a-3p; FDR = 0.02). A list of interacting DE miRNAs in RB sEVs with dysregulated target cell cycle-related genes including c-MYC and MYCN were listed in Table 3.

**Figure 4:**
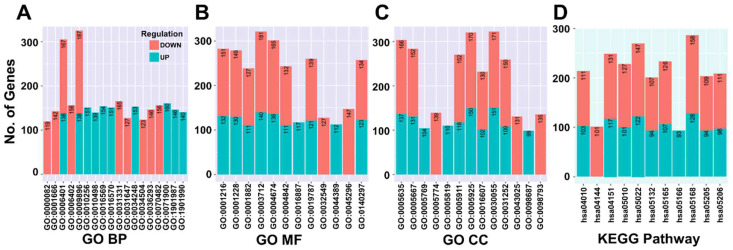

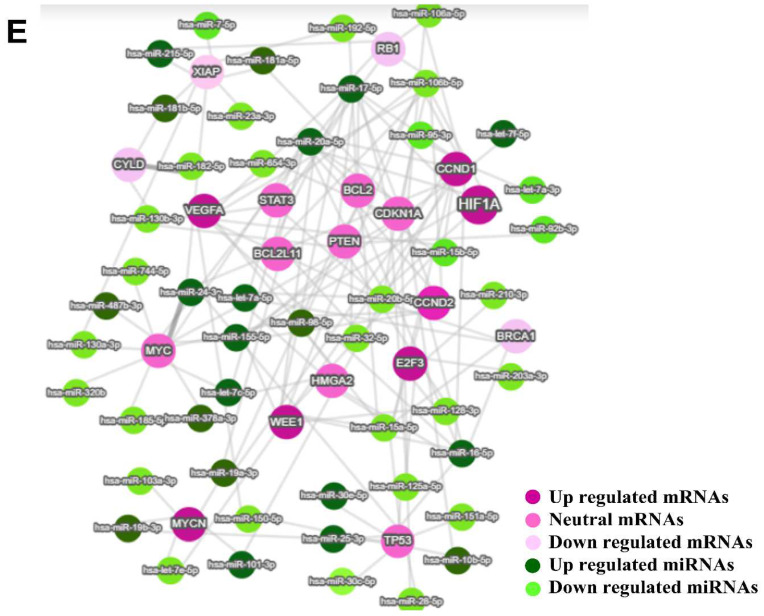
Functional enrichment analysis of differentially expressed miRNA-targets in Retinoblastoma (RB) serum small EVs. (**A**–**C**). Gene ontology (BP—Biological process, MF-Molecular function and CC—Cellular component) terms (**D**). KEGG terms. (**E**): The interaction network of differentially expressed (DE) miRNAs and their experimentally validated target genes (DE mRNAs) identified in retinoblastoma serum small EVs was generated by miRTargetLink 2.0 using the strong interaction and minimum 5 shared targets options.

The significance of DE miRNA targets in RB was determined by functional enrichment analysis. The top GO and KEGG terms were represented in bar graphs (Figure 4A–D). Up regulated miRNA targets are associated with regulation of protein serine/threonine kinase activity (GO:0071900), covalent chromatin modification (GO:0016569), and down regulated miRNA targets are related to positive regulation of cellular catabolic process (GO:0031331). Both up and down miRNA targets were enriched with RNA catabolic process (GO:0006401), and transcription co-regulator activity (GO:0003712), MAPK signaling pathway (hsa04010), PI3K-Akt signaling pathway (hsa04151), and proteoglycans in cancer (hsa05205).

### 3.6. miRNA-mRNA Regulatory Network Results

Based on the miRNA enrichment results, the top DE miRNAs having the highest targets and mRNAs targeted by maximum number of DE miRNAs were used as input for constructing experimentally validated miRNA–target interaction (MTI) network. This co-expression network unveiled the expression status of 35 miRNAs and their targets 19 mRNAs in RB sEVs (Figure 4E). A total of 562 functional MTI pairs were generated (Supplementary File S1). The key DE mRNAs VEGFA, CCND1, E2F3, WEE1, c-MYC, HIF1A, and XIAP in the network were found to be regulated by highly dysregulated miRNAs such as 16-5p, 106a-5p, 24-3p, 17-5p, 15a-5p, 181a-5p, 181b-5p, and 15b-5p. miRNA-mRNA network enrichment results revealed that the target genes in the network are linked to pathways in cancer, microRNAs in cancer and cell cycle (KEGG, *p* < 0.05), Cyclin D associated events in G1, ubiquitin specific processing proteases (reactome pathways, *p* < 0.05), PI3K-Akt Signaling, G1 to S cell cycle control and retinoblastoma gene in cancer (wiki pathways, *p* < 0.05).

### 3.7. DE lncRNA Analysis and Functional Enrichment Analysis of Target Genes

**Figure 5:**
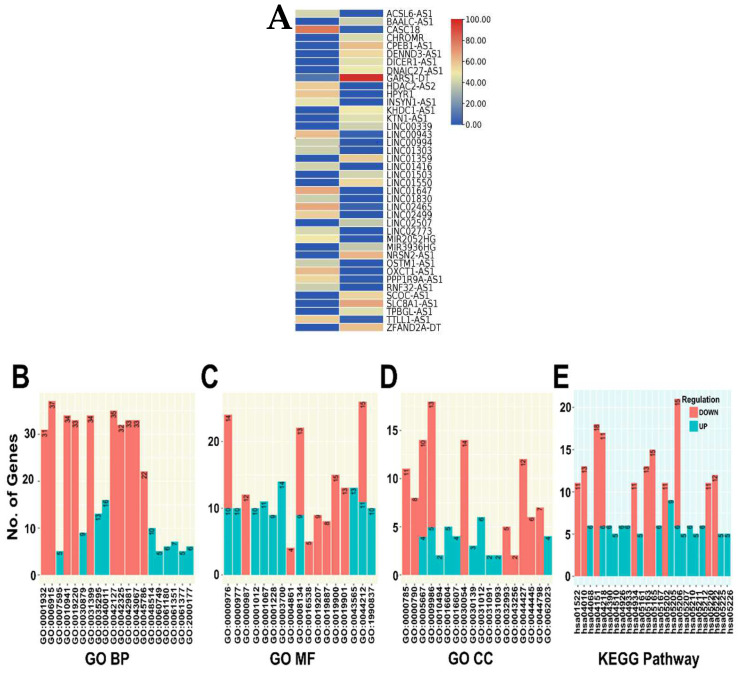

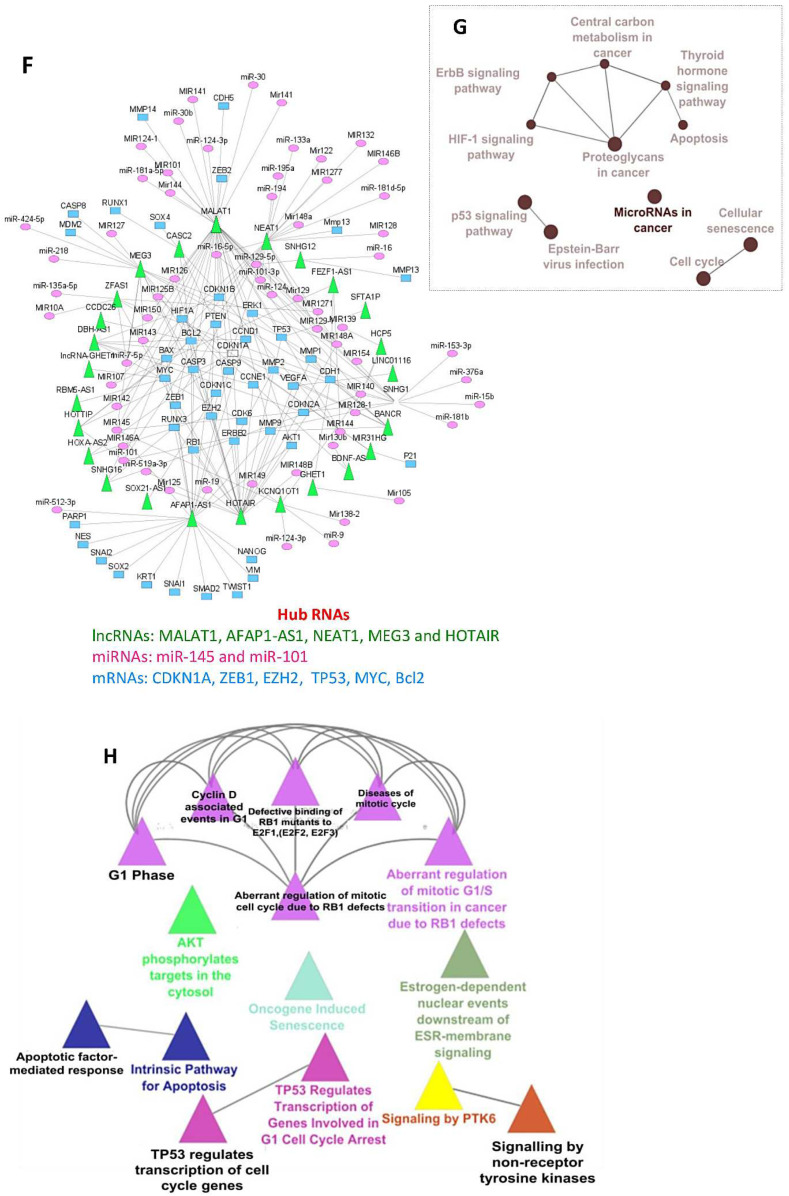
(**A**) Heat map of differentially expressed lncRNAs in retinoblastoma serum small extracellular vesicles. Functional enrichment of lncRNA-target genes: (**B**–**D**). Gene ontology (BP- Biological process, MF-Molecular function and CC- Cellular component) analysis and (**E**). Kyoto Encyclopedia of Genes and Genomes (KEGG) enrichment results. (**F**) lncRNA-miRNA-mRNA interactions in retinoblastoma serum-derived small extracellular vesicles. Selected enriched (**G**) KEGG terms and (**H**). GO-Biological processes terms for protein-coding mRNAs in the network.

The majority of the DE lncRNAs identified in RB sEVs, including the top ones, belong to long intergenic non-coding RNAs (LINCRNAs): LINC02499, LINC02773, LINC01416 and LINC00994, whereas OXCT1-AS1, HDAC2-AS2, ACSL6-AS1, and SLC8A1-AS1 are some of the antisense DE lncRNAs that are able to control their own sense gene expression. The DE lncRNAs are represented in a heat map (Figure 5A). LncRNA target gene analysis revealed that of 3634, only 242 lncRNAs in RB sEVs have targets. A total of 769 regulatory lncRNA-miRNAs pairs, and 541 lncRNA-mRNA pairs comprised of 242 lncRNAs, 323 miRNAs, and 332 mRNAs were predicted (Supplementary File S2). The significantly up regulated AFAP-AS1, and down regulated MALAT1, GAS5, ZFAS1, and SNHG16 have the highest number of target interactions. In addition, lncRNAs that directly target cell cycle specific genes were also detected in RB sEVs (Table 3).

**Table 3:** Expression status and interactions of miRNAs and lncRNAs with their target dysregulated cell cycle specific genes identified in RB serum small extracellular vesicles.

Cell Cycle Specific Genes Dysregulated in RB Serum Small EVs	Fold Change	FDR	No. of Interacting miRNAs (Up Regulated) in RB Serum Small EVs	No. of Interacting miRNAs (Down Regulated) in RB Serum Small EVs	No. of Interacting lncRNAs (Up/Down/N (Neutral) in RB Serum Small EVs
RB1	−6.6	0.007	4 (17-5p, 20a-5p, 132-3p, 215-5p)	6 (23b-3p, 106b-5p, 192-5p, 130b-3p, 221-3p, 20b-5p)	HOTAIR (N), AATBC (N), MEG3 (N), RB1-DT (N), and PANTR1 (N)
CCND1	4.03	0.005	14 (20a-5p, 16-5p, 19a-3p, 17-5p, 425-5P, 155-5p, 24-3p, let-7f-5p, let-7c-5p, let-7a-5p, 98-5p, 101-3p, 342-5p)	10 (15a-5p, 15b-5p, 106b-5p, 142-5p, 340-5p, 20b-5p, 7706, 323b-3p, let-7e-5p, 7a-3p)	AFAP1-AS1 (Up), DBH-AS1 (Up), MALAT1 (Down)
E2F3	2.03	0.01	7 (17-5p, 20a-5p, 101-3p, 24-3p, 16-5p, 660-5p, 425-5P)	16 (210-3p, 128-3p, 106b-5p, 203a-3p, 221-3p, 32-5p, 30c-5p, 15a-5p, 15b-5p, 92b-3p, 103b, 20b-5p, 4732-3p, 423-5p, 199a-5p, 125a-5p)	FLVCR1-DT (N), NORAD (N) and NEAT1 (N)
CDKN1A	−2.2	1.0	14 (182-5p, 20a-5p, 17-5p, 132-3p, 146b-5p, 10b-5p, 98-5p, let-7f-5p, 7a-5p, 16-5p, 7c-5p, 101-3p, 133a-3p, 181a-5p)	11 (654-3p, 363-3p, 345-5p, 28-5p, 20b-5p, 125a-5p, 106b-5p, 15a-5p, 15b-5p, 148b-3p, let-7e-5p)	HOTAIR (N), BANCR (Up), DBH-AS1 (Up), HOSA-AS2 MALAT1 (Down), SNHG1 (Down), HOTTIP (Down) MIR31H1G (Down)
CDKN1B	−7.6	7.5× 10^−5^	181a-5p, 24-3p, 155-5p, 182-5p,	148-5p	DBH-AS1 (Up) and MALAT1 (Down)
TP53	−1.9	1.0	11 (16-5p, 10b-5p, 324-5p, 150-5p, 30e-5p, 19b-3p, 20a-5p, 17-5p, 19a-3p, 24-3p, 330-3p)	10 (125a-5p, 25-3p, 15a-5p, 221-3p, 30c-5p, 106b-5p, 185-5p, 3529-3p, 151a-5p, 28-5p)	MALAT1 (Down) MEG3 (N) SFTA1P (Down), and SNHG1(Down)
c-MYC	1.54	1.0	14 (24-3p, 98-5p, 155-5p, 17-5p, 20a-5p, 378a-3p, 487b-3p, 19a-3p, 16-5p, 148a-5p, 29a-3p, let-7a-5p, 7c-5p, 7f-5p)	18 (320b, 744-5p, 423-5p, 323a-3p, 16-2-3p, 7-5p, 126-5p, 25-3p, 106b-5p, 92b-3p, 30c-5p, 23a-3p, 196b-5p, 151a-5p, 125a-5p, let-7e-5p, 130a-3p, 185-5p)	AFAP1-AS1 (Up), PVT1 (N), MALAT1 (Down), RBM5-AS1 (Down), PCATC (N)
MYCN	6.8	0.003	4 (101-3p, 29a-3p, 19b-3p, 19a-3p)	4 (let-7e-5p, 126-5p, 144-3p, 103a-3p)	-
MDM2	−6.6	0.007	6 (17-5P, 20a-5p, 330-3p, 29a-3p, 381-3p, 425-5p	13 (32-5p, 25-3p, 143-3p, 221-3p, 92b-3p, 363-3p, 20b-5p, 106b-5p, 185-5p, 59, let-7a-3p, 339-5p, 340-5p	MEG3 (N)
WASL	−6.9	0.002	8 (17-5p, 98-5p, let-7f-5p, let-7c-5p, let-7a-5p, 20a-5p, 19a-3p, 19b-3p)	15 (27b-3p, 379-5p, 148b-3p, 128-3p, 323a-3p, let-7e-5p, 363-3p, 92b-3p, 32-5p, 25-3p, 130b-3p, 130a-3p, 20b-5p, 106b-5p, -590-3p)	SNHG14 (N) and CDKN2B-AS1 (N)
HSP90AA11	−7.4	0.0002	7 (16-5p, 425-5p, 421, 378a-3p, 30e-5p, 17-5p, 101-3p)	7 (760, 185-5p, 30c-5p, 25-3p, 889-3p, 148b-3p, 23a-3p)	-
XIAP	−7.6	0.0001	13 (181a-5p, 181b-5p, 215-5p, 101-3p, 17-5p, 24-3p, 20a-5p, 421, 122-5p, 150-5p, 10b-5p, 19b-3p, 19a-3p)	13 (192-5p, 7-5p, 106b-5p, 20b-5p, 130a-3p, 584-5p, let-7e-5p, 889-3p, 143-3p, 15b-3p, 451b, 130b-3p, 23a-3p)	AFAP1-AS1 (Up), DANCR (N), GHET1 (Down), MALAT1 (Down), PCAT6 (N), PCGEM1 (N), PVT1 (N), and RBM5-AS1 (Down)
AKAP8	−8.1	6.1 × 10^−6^	5 (146b-5p, let-7f-5p, let-7c-5p, let-7a-5p, 98-5p)	2 (92b-3p, let-7e-5p)	-
BRCA1	−2.2	0.006	5 (16-5p, 24-3p, 215-5p, 181a-5p, 10b-5p)	3 (15a-5p, 192-5p, 20b-5p)	-
CYLD	−2.3	0.009	5 (17-5p, 16-5p, 20a-5p, 181b-5p, 182-5p)	6 (106b-5p, 20b-5p, 15b-5p, 15a-5p, 130b-3p, 126-5p)	-
FBXO31	−6.9	0.002	4 (17-5p, 20a-5p, 3074-5p, 10b-5p)	7 (192-5p, 92b-3p, 106b-5p, 339-5p, 451b, 3529-3p, 20b-5p)	-
KIF2C	2.5	0.001	6 (101-3p, 16-5p, 20a-5p, 181a-5p, 181b-5p, 181c-5p)	2 (148b-3p, 142-5p)	-
CEP55	7.1	0.005	5 (155-5p, 215-5p, 16-5p, 19a-3p, 19b-3p)	10 (192-5p, 103a-3p, 130a-3p, 130b-3p, 148b-3p, 15a-5p, 15b-5p, 411-5p, 199b-3p, 199a-3p)	-

The enriched GO and KEGG terms for lncRNA-target genes are depicted in bar graphs (Figure 5B–E). The down regulated lncRNA-target genes are correlated with the apoptotic process (GO:0006915), regulation of cell proliferation (GO:0042127), regulation of phosphorylation (GO:0042325) regulation of apoptotic process (GO:0042981), transcription regulatory region DNA binding (GO:0044212), PI3K-Akt signaling pathway (hsa04151), and microRNAs in cancer (hsa05206). 

### 3.8. LncRNA-miRNA-mRNA Network Results

To reveal the regulatory role of lncRNAs on miRNAs and protein-coding genes associated with RB tumorigenesis, a lncRNA-miRNA-mRNA network consisting of 23 lncRNA, 64 miRNA, and 46 mRNA nodes with 203 edges (101 lncRNA-miRNA pairs and 102 lncRNA-mRNA pairs) was constructed (Figure 5F and Supplementary File S3). We found that the protein-coding genes targeted by lncRNAs and miRNAs in the network were associated with the cell cycle, such as RB1, CDK6, cyclins (CCND1 and CCNE1), CDK inhibitors (CDKN1A, CDKN1B, CDKN1C and CDKN2A). Based on the degree, closeness and betweenness centrality, the hub lncRNAs (MALAT1, AFAP1-AS1, HOTAIR, NEAT1 and MEG3), miR145 and miR 101, and mRNAs (CDKN1A, EZH2 and ZEB1) were identified from network analysis (Table 4). Hub RNAs was found to be involved in more regulatory interactions having key roles in network organization. The CluGo enriched functional KEGG terms for protein coding genes in the network are cell cycle, apoptosis and tumor related signaling pathways such as HIF1A, ErbB, and P53 (Figure 5G). In addition, the most relevant biological process related to RB “aberrant regulation of mitotic G1/S transition in cancer due to RB1 defects” was also enriched for target mRNAs in the network (Figure 5H). These results suggest that lncRNAs play a crucial role in mediating RB progression.

### 3.9. Quantitative Reverse Transcriptase-Polymerase Chain Reaction Validation

The mRNA expression levels of HIF1A (hypoxia-inducible factor 1-alpha), PGK1 (phosphoglycerate kinase 1), and SYK (spleen associated tyrosine kinase) were tested in five primary RB tumors and two control retinas by RT-qPCR. There was a statistically significant (*p* < 0.05) overexpression of SYK in three RB tumors and HFIA in one RB tumor compared to control retina (Figure S4). However, the expression levels of PGK1 remained same in RB tumors and control retina.

## 4. Discussion

Serum-derived small extracellular vesicles (sEVs) can be a possible potential source of liquid biopsy for tumors such as RB, where routine tissue biopsy for diagnosis is not done due to the risk of extraocular tumor spread [48]. In this comprehensive study, we characterized RB serum sEVs coding and non-coding RNA content, and explored the miRNA-mRNA, and lncRNA-miRNA-mRNA regulatory interactions. Until now, only one previous study had analyzed small non-coding miRNAs in serum sEVs of RB patients using NGS technology [21]. 

An average of 5–6 × 10^11^ particles/mL, with size 120–135 nm, and ZP of 11.0–12.6 mV was recovered from RB and non-RB serum samples. There was no difference in sEV concentration detected between the two groups. However, smaller size and lower ZP values were observed for single freeze-thaw cycled RB sEVs compared to non-RB. All the isolated serum sEV samples were positive for well-known exosome markers CD9, CD81, and TSG 101 [49]. To our knowledge, the physical properties of sEVs of young children (<5 years) have not yet been reported. However, studies on adult sEVs revealed that they have a diameter of 109 ± 4 nm, 1.8 × 10^11^ ± 3.1 particles/mL and ZP of −9.80 to −21.1 mV [50]. Another group documented the presence of 1–3 × 10^12^ sEVs/1 mL in adult serum [51]. Physico-chemical properties of sEVs can provide insights about intricate pathological processes in the course of the disease and have been evolving as potential factors in cancer diagnosis and monitoring treatment response [5,7,9]. Elevated levels of sEVs have been found in cancer patients compared to healthy donors [52,53,54]. Plasma membranes of cells have a negative surface charge, which is known to influence various biological processes such as cellular uptake and cytotoxicity [25]. Zeta potential (ZP), an indicator of colloidal stability, is influenced by the surface charge. The difference in ZP of RB and non-RB sEVs could be due to aberrant biological processes in RB patients. The reason for smaller size of RB sEVs compared to non-RB patients is not known. The literature states that the difference in vesicle size is not a crucial criterion, as sEVs might aggregate into larger vesicles, or multi-vesicular bodies can split into smaller vesicles. Moreover, it remains unknown whether one cell produces EVs of different sizes, if the difference in size reflects EV production by different cells, or whether vesicles with a common size share the same or different composition [55]. 

Our whole transcriptome analysis on large RNA and small RNA seq on small RNA fractions recovered from the same samples disclosed the presence of diverse RNA cargoes in sEVs. However, the proportions of individual RNA species varied between RB and non-RB groups. The expression profiles revealed up regulation of 2434 mRNA, 1474 lncRNAs, and 41 miRNAs, and down regulation of 4080 mRNAs, 2160 lncRNAs, 82 miRNAs in RB sEVs compared to non-RB sEVs. Consistent with previous microarray and RNA seq gene expression data on primary RB tumor tissues [56,57,58], candidate coding (RB1, E2F3, MYCN, MDM2, KIF14, MDM4, DEK, CDH11, CEP170, SIX1, SIX4, and SYK) and ncRNAs (miR-17-5p, 20a-5p, 324-5p, 182-5p, 181a-3p, 191-3p, 451b, hsa-let 7a, let 7e,) involved in RB tumorigenesis were also found to be dysregulated in RB sEVs. Elevated expression profiles of miR-17-5p, 20a-5p, 215-5p, 16-5P, 150, 155, and low levels of let (7e-5p, 7d), miR15a, 15b and 106b-5p were found in RB sEVs and were consistent with their expression in RB tissues. However, the expression status of miR-29a, 98-5p, 7f-5p, 133a-3p, 330-3p, 101-3p, 25-3p, and 143 in RB sEVs from our study showed reverse correlation with available RB tissue datasets [59,60,61]. Interestingly, RB sEV miRNA expression profiles form our study correlated well with previous RB serum vs. control serum miRNA profiles by Beta et al. [62] (Table S9). Previous RB sEVs miRNA data revealed that except 301b-3p and 216b-5p, the miRNA expression in sEVs and corresponding RB tumor tissue samples were not matching [21]. Interestingly, these two miRNAs were not expressed in our samples. Highly aberrant lncRNAs detected in our RB sEVs OXCT1-AS1, HPYR1, HDAC2-AS2, LINC02499, slc8a1-as1, ZFAND2A-DT, LINC01359, SCOC-AS1 have not yet been reported in RB. Except AFAP1-AS1 and BANCR, all other RB sEVs lncRNA (BDNF-AS, MALAT1, HOTAIR, PANDAR, XIST, DANCR, UCA1, ZFPM2-AS1, SNHG16, and NEAT1) expression patterns from the present study were not replicating with RB tissues [63,64,65,66,67,68,69,70,71,72,73,74,75,76,77] (Table S10). The distinct RNA expression profiles in sEVs and corresponding tumor tissues, among different RB patients, and from different cohorts, could be due to intra and inter-individual variability as well as tumor heterogeneity. In addition, as serum is a body fluid in a systemic situation, RB sEVs possibly contain molecular cargo from all body cell types. Apart from this, there is a potential chance of miRNAs contaminants from other sources/or produced as a result of various cellular events such as in response to stress and inflammation or active secretion by a protein-miRNA complex (e.g., high-density lipoprotein: HDL) and argonaute protein (e.g., Ago2) [78].

Identification of the most commonly deregulated genes for a specific cancer is of potential diagnostic interest. Several studies have reported that tumor-derived exosomes from several cancers reflect original tumor molecular signatures, and they share distinct transcriptome and proteomic profiles with respect to healthy controls [7,79]. However, high throughput data of non-coding RNAs related to RB as well as RB EVs are not available in the literature. A large number of RB patients’ sEVs, and corresponding RB tumors must be analyzed to obtain a sound conclusion. GO, KEGG and GSEA analysis of DE mRNAs, DE lncRNA-target genes, and DE miRNA target genes were significantly enriched in biological processes related to angiogenesis, EMT, mitotic cell cycle regulation, chromatin organization, metabolic pathways, and with cancer-related pathways, such as the PI3K-Ak signaling, TGF-beta signaling, p53 signaling, Insulin signaling, and HIF1 signaling pathways. 

PPI network analysis of 39 DEMs related to eye and retina development were shown to be involved in biological processes associated with 9-cis-retinoic acid (RA) biosynthetic processes, retinal rod cell differentiation and retinal cone cell development. Surprisingly, cone specific phosphodiesterase 6C (PDE6C) expression, which is restricted to the retina, pineal gland, and retina-derived tumors [80], was also detected in sEVs. However, their levels were high in normal sEVs compared to RB. The presence of PDE6C in sEVs indicates that eye EVs can enter into the circulation system. In support of our findings, previous studies reported that EVs can cross the blood-retinal barrier (BRB) and blood-brain barrier (BBB) and serve as a source of communication between the central nervous system (CNS) and peripheral system [81]. In addition, consistent with our data, earlier studies reported that RA induces cone-specific and rod-specific gene inactivation, and cell cycle arrest during the differentiation of RB cells, which implicates the role of RA signaling in RB development [82]. Moreover, NGS data of normal retinas, RB tumors and RB cell lines revealed that abnormal retina development could be involved in RB origin and progression [83,84].

Exploration of regulatory interactions between ncRNA-target mRNAs is crucial for elucidating ncRNA-mediated gene regulation in RB, as emerging data has provided evidence that epigenetic molecular events driven by RB1 loss are necessary for malignant phenotype [85]. We identified several miRNAs (miR 101-3P, 16-5p, 155-5P, 181a-5p, miR17/192, LET-7 clusters, and lncRNAs (HOTAIR, KCNQ1OT1, MALAT1, AFAP-S1) that directly target common dysregulated cell cycle genes in RB including RB1, CCND1, E2F3, WEE1, XIAP, CDKN1A, c-MYC, MYCN, BCL2, VEGEF, HIF1A, and P53. Although mutations in RB1 gene are responsible for most cases of RB, identification of miRNAs and lncRNAs that directly target RB1 in unilateral sporadic cases with RB1^+^/^+^ genotype tumors aid in uncovering novel non-genetic mechanisms of RB1 inactivation. Similar to our findings, previous studies reported that miRNAs, and lncRNAs negatively regulate translation of target cell cycle-related gene cyclins, E2F members, cell cycle inhibitors, and TGFB2 [86,87,88]. However, in vitro and in vivo functional studies must be done to validate/confirm the individual coding-noncoding interactions identified in RB sEVs. Along with this, screening these genes in sEVs and corresponding tumor from different cohorts of RB patients would reveal potential EV diagnostic markers.

Based on in silico analysis and functional studies, several studies have investigated regulatory interactions between coding and ncRNAs in RB [89,90,91]. However, in most cases, only ncRNAs and their target genes that are aberrantly expressed in other cancers were selectively picked up and studied for their role in RB. However, the detailed analysis of direct interactions between mRNA-ncRNA regulatory axis has not been much explored as miRNA and lncRNA have very short lifespans and their expression pattern is very dynamic in a given cell. These properties make it difficult to understand ncRNA regulation by wet lab experiments in a single study. Computational analysis of lncRNA-miRNA-mRNA interactions would provide a theoretical basis for the molecular mechanism of disease, and for the identification of potential therapeutic targets [92]. However, to our knowledge only a single study has demonstrated autophagy-related lncRNA-miRNA-mRNA regulatory networks in RB [93]. Thus, we investigated the direct interactions between lncRNA-miRNA, miRNA-mRNA and lncRNA-mRNA, and constructed lncRNA-miRNA-mRNA network. Based on the degree, closeness and betweenness centrality, MALAT1, HOTAIR, NEAT1, AFAP1-AS1, miR 145, and miR101 were identified as hub ncRNAs that play central roles in RB pathogenesis. Target protein coding genes for these hub ncRNAs are associated with cell cycle (RB1, c-MYC, cyclins, CDKs), cellular senescence, remodeling of extracellular matrix (MMP1, MMP2, and MMP9), and epigenetics (EZH2 and ZEB1). PI3k-AKT, HIF1, ErbB, and P53 signaling pathways are enriched terms. These are the known signaling pathways that drive RB tumorigenesis [57,94,95]. The hub genes EZH2 and ZEB are well-studied epigenetic regulators in RB. ZEB1 is an EMT transcription factor, usually repressed by RB1. This facilitates the epigenetic silencing of CDH1 (E-cadherin). The XIST-miR101-ZEB1 axis has been shown to be responsible for malignant properties of RB cells [70]. Our RT-qPCR data showed a high expression of epigenetic regulator, SYK and hypoxia-inducible transcription factor, HIF1A in RB tissues compared to control retinas. However, the expression status of SYK and PGK1 in RB sEVs and corresponding RB tissues were not matching. It is worth noting that the study included low number of sEV RNA samples sequenced per group (*n* = 3), and the presence of minor contaminants of serum in the analyzed sEV data due to technical challenges of EV extraction with the available commercial kits.

## 5. Conclusions

The serum EV RNA profiling in non-invasive RB tumors suggests that sEVs have the signatures of RB tumors. The cargo in the small EVs is involved in the epigenetic regulation of cell cycle, metabolism, and tumor-associated signaling pathways. Further validation is warranted to use them as prognostic biomarkers.

## Figures and Tables

**Table 1:** Upregulated Retinoblastoma serum-derived small extracellular vesicles genes involved in ocular development.

S No	Gene	Function	Log2 (Fold Change)	FDR
1	PAX4 (Paired Box 4)	Retina development in camera type eye	4.5	3.1× 10^−7^
2	WNT5A (Wnt Family Member 5A)	Optic cup formation involved in camera type eye development	8.5	3.9× 10^−7^
3	INHBA (Inhibin Subunit Beta A)	Eyelid development in camera type eye	3.4	0.0001
4	PFDN5 (Prefoldin Subunit 5)	Retina development in camera type eye	7.4	0.0003
5	RARB (Retinoic Acid Receptor Beta)	Embryonic eye morphogenesis	2.8	0.0004
6	RBP4 (Retinol Binding Protein 4)	Eye development	7.3	0.0005
7	ALDH1A2 (Aldehyde Dehydrogenase 1 Family Member A2)	Embryonic camera type eye development	2.6	0.0006
8	TWSG1 (Twisted Gastrulation BMP Signaling Modulator 1)	Camera type eye development	4.42	0.001
9	BHLHE23 (Basic Helix-Loop-Helix Family Member E23)	Post embryonic eye morphogenesis	7.1	0.001
10	MEIS3 (Meis Homeobox 3)	Eye development	3.1	0.001
11	TULP1 (TUB Like Protein 1)	Retina development in camera type eye	2.5	0.001
12	OLFM3 (Olfactomedin 3)	Eye photoreceptor cell development	2.7	0.002
13	CYP1B1 (Cytochrome P450 Family 1 Subfamily B Member 1)	Retina vasculature development in camera type eye	6.9	0.002
14	PDE6B (Phosphodiesterase 6B)	Retina development in camera type eye	3.0	0.002
15	PTN (Pleiotrophin)	Retina development in camera type eye	2.4	0.01
16	PROX1 (Prospero Homeobox 1)	Retina morphogenesis in camera type eye	2.7	0.01
17	CYP1A1 (Cytochrome P450 Family 1 Subfamily A Member 1)	Camera type eye development	2.9	0.01
18	ACHE (Acetylcholinesterase)	Retina development in camera type eye	2.1	0.01
19	PDGFRA (Platelet Derived Growth Factor Receptor Alpha)	Retina vasculature development in camera type eye	2.2	0.04
20	BMPR1B (Bone Morphogenetic Protein Receptor Type 1B)	Retina development in camera type eye	2.5	0.05

**Table 2:** Down regulated Retinoblastoma serum-derived small extracellular vesicles genes involved in ocular development.

S No	Gene	Biological Function	Log2 (Fold Change)	FDR
1	SOX8 (Sex Determining Region Y) Transcription Factor 8)	Negative regulation of photoreceptor cell differentiation, Retina development in camera type eye	−7.4	0.0003
2	SPATA7 (Spermatogenesis Associated 7)	Photoreceptor cell maintenance	−7.2	0.0007
3	OPN3 (Opsin 3)	Phototransduction	−5.9	0.04
4	PDE6C (Phosphodiesterase 6C)	Phototransduction visible light	−6.7	0.005
5	RP1 (Retinitis Pigmentosa 1 Axonemal Microtubule Associated)	phototransduction, visible light, Retina development in camera type eye	−3.9	0.0001
6	IFT20 (Intraflagellar Transport 20)	Photoreceptor cell outer segment organization	−7.2	0.0005
7	BAK1 (BCL2 Antagonist/Killer 1)	Post embryonic camera type eye morphogenesis	−6.5	0.01
8	CTNS (Cystinosin, Lysosomal Cystine Transporter)	Lens development in camera-type eye	−7.0	0.001
9	PAX2 (Paired Box 2)	Optic cup morphogenesis involved in camera type eye development	−4.6	0.0001
10	GNB1 (G Protein Subunit Beta 1)	Retina development in camera-type eye	−7.8	3.1× 10^−5^
11	CRYBG3 (Crystallin Beta-Gamma Domain Containing 3)	Lens development in camera type eye	−7.5	0.0001
12	XRN2 (5′-3′ Exoribonuclease 2)	Retina development in camera type eye	−6.8	0.003
13	YY1 (Transcription Factor)	Camera type eye morphogenesis	−7.8	4.3× 10^−5^
14	BMP7 (Bone Morphogenetic Protein 7)	Embryonic camera type eye morphogenesis	−2.8	0.005
15	HSF4 (Heat Shock Transcription Factor 4)	Camera type eye development	−7.2	0.0007
16	CALB1 (Calbindin 1)	Retina development in camera type eye	−6.8	0.003
17	PBX4 (PBX Homeobox 4)	Eye development	−5.9	0.04
18	SLC1A1 (Solute Carrier Family 1 Member 1)	Retina development in camera type eye	−6.5	0.01
19	GATA3 (GATA Binding Protein 3)	Lens development in camera type eye	−7.1	0.001

**Table 4:** Details of hub genes identified from lncRNA-miRNA-mRNA network.

Gene	Closeness Centrality	Betweenness Centrality	Degree Layout
MALAT1	0.45	0.5	42
HOTAIR	0.4	0.22	25
NEAT1	0.35	0.2	24
AFAP1-AS1	0.36	0.2	23
MEG3	0.35	0.1	15
SNHG1	0.34	0.1	13
CDKN1A	0.36	0.08	8
MIR145	0.39	0.08	6
EZH2	0.37	0.04	5
ZEB1	0.39	0.06	5 MIR101 0.31 0.02 4 BCL2 0.33 0.05 6 TP53 0.36 0.04 5

## Data Availability

The datasets used and/or analyzed during the current study are available from the corresponding author on reasonable request.

## Supplementary Materials

The following supporting information can be downloaded at: https://www.mdpi.com/article/10.3390/cancers14174179/s1. Table S1: Primers for RT-qPCR. Table S2: Clinical characteristics of subjects included in the study. Table S3: The concentration, size distribution and zeta potential of serum small extracellular vesicles from retinoblastoma (RB) and non-RB subjects. Table S4: RNA sequencing details of serum small EVs from RB and non-RB subjects. Table S5: Top 20 up regulated mRNAs in RB serum small EVs. Table S6: Top 20 down regulated mRNAs in RB serum small EVs. Table S7: Top 20 up regulated miRNAs in RB serum small EVs. Table S8: Top 20 down regulated miRNAs in RB serum small EVs. Table S9: Dysregulated microRNAs identified in RB serum small EVs and their expression in RB tumor tissues from review of literature. Table S10: Dysregulated lncRNAs identified in retinoblastoma (RB) serum small EVs and their expression in RB tumor tissues from a review of the literature. Figure S1: Research flow chart. Figure S2: Protein-protein interaction network of differentially expressed mRNAs of RB serum small EVs related to eye development. Figure S3: miRNA-target interactions in RB serum small EVs. Figure S4: Real time expression data of HIF1A, PGK1, and SYK File S1: Exosomal miRNA-mRNA network in RB. File S2: RB small EV lncRNAs and lncRNA targets, and File S3: RB exosomal lncRNA-miRNA-mRNA network.
