# Supplementary material for: Comprehensive Analysis of Serum Small Extracellular Vesicles-Derived Coding and Non-Coding RNAs from Retinoblastoma Patients for Identifying Regulatory Interactions
Source: Cancers (Basel). 2022 Aug 29;14(17):4179. doi: 10.3390/cancers14174179 (PMC9454787; doi:10.3390/cancers14174179)
Supplement: Supplementary file 1 [file cancers-14-04179-s001.zip › cancer-1828690 - Supplementary Figures.pdf]

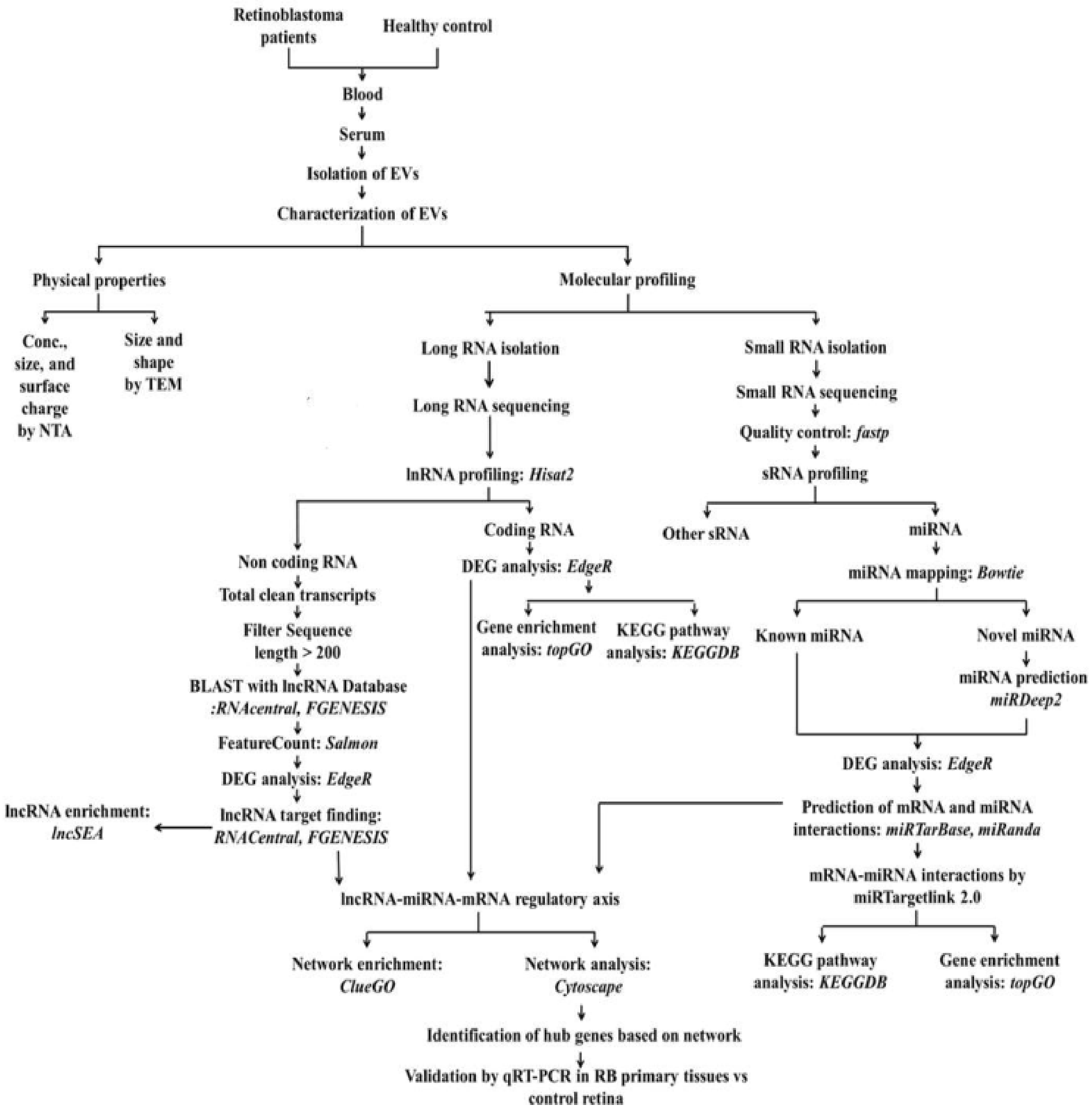

Figure S1. Research work flow.

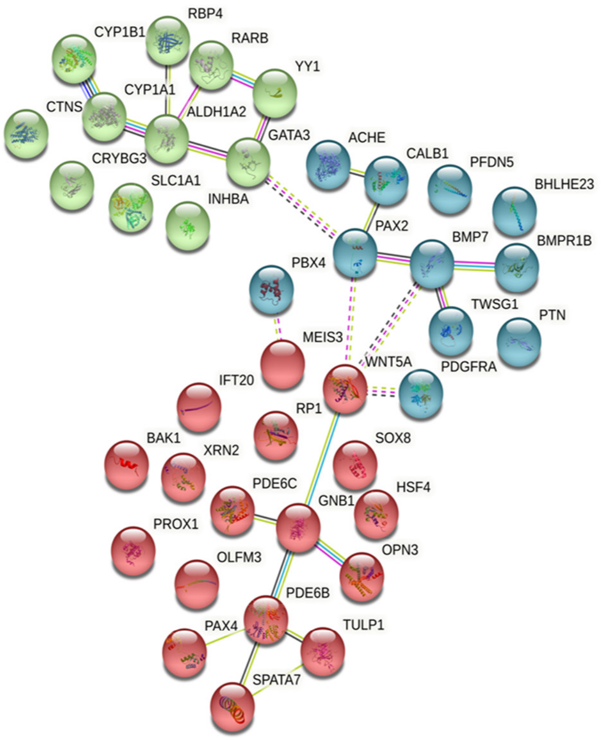

CLUSTER 1: 11 genes
PPI enrichment p-value: 1.39e-05
Biological processes
- 9-cis-retinoic acid biosynthetic process (P=0.003)
- Retinol metabolic process (FDR=3.58e-05)
- Camera-type eye development (FDR=2.00e-11)
- Secondary metabolic process (FDR=0.002)
- Lens development in camera-type eye (FDR=0.005)

CLUSTER 2: 11 genes
PPI enrichment p-value: 0.0007
Biological processes
- Embryonic camera-type eye morphogenesis (FDR=0.02)
- Retina development in camera-type eye (FDR=1.20e-09)
- Camera-type eye morphogenesis: (FDR=0.001)
- Camera-type eye development: (FDR=2.00e-11)
- Mesoderm formation (FDR=0.004)

CLUSTER 3: 17 genes
PPI enrichment p-value: 0.0003
Biological processes
- Protein localization to photoreceptor outer segment (FDR=0.01)
- Phototransduction, visible light (FDR=2.24e-06)
- Retinal rod cell differentiation (FDR=0.02)
- Retinal cone cell development (FDR=0.02)
- Camera-type eye photoreceptor cell differentiation (FDR=0.001)
- Photoreceptor cell differentiation(FDR=2.47e-08)

Uniprot annotated keywords
KW-0844: Vision (FDR= 7.75e-05)
KW-0716: Sensory transduction (FDR=0.02)
KW-0682: Retinitis pigmentosa (FDR=0.01)
KW-0238: DNA-binding (FDR=0.02)
KW-0225: Disease mutation(FDR=0.02)

Disease gene associations: DOID:5614 Eye disease (FDR=0.0003)

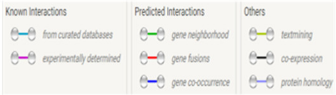

Figure S2. Protein-protein interaction (PPI) network of differentially expressed (DE) mRNAs of RB serum exosomes related to eye development. The network was constructed using string version: 11.5. K-means clustering with minimum 3 clusters settings were applied. Three clusters were generated with 11 genes in cluster 1 (green bubbles), 11 genes in cluster 2 (blue bubbles) and 9 genes in cluster 3 (red bubbles). The selected significant biological functions for each cluster with FDR values were given. Network status: Number of nodes: 39; Number of edges: 25; Average node degree: 1.28; Avg. local clustering coefficient: 0.35; PPI enrichment p-value: 4.1e-05.

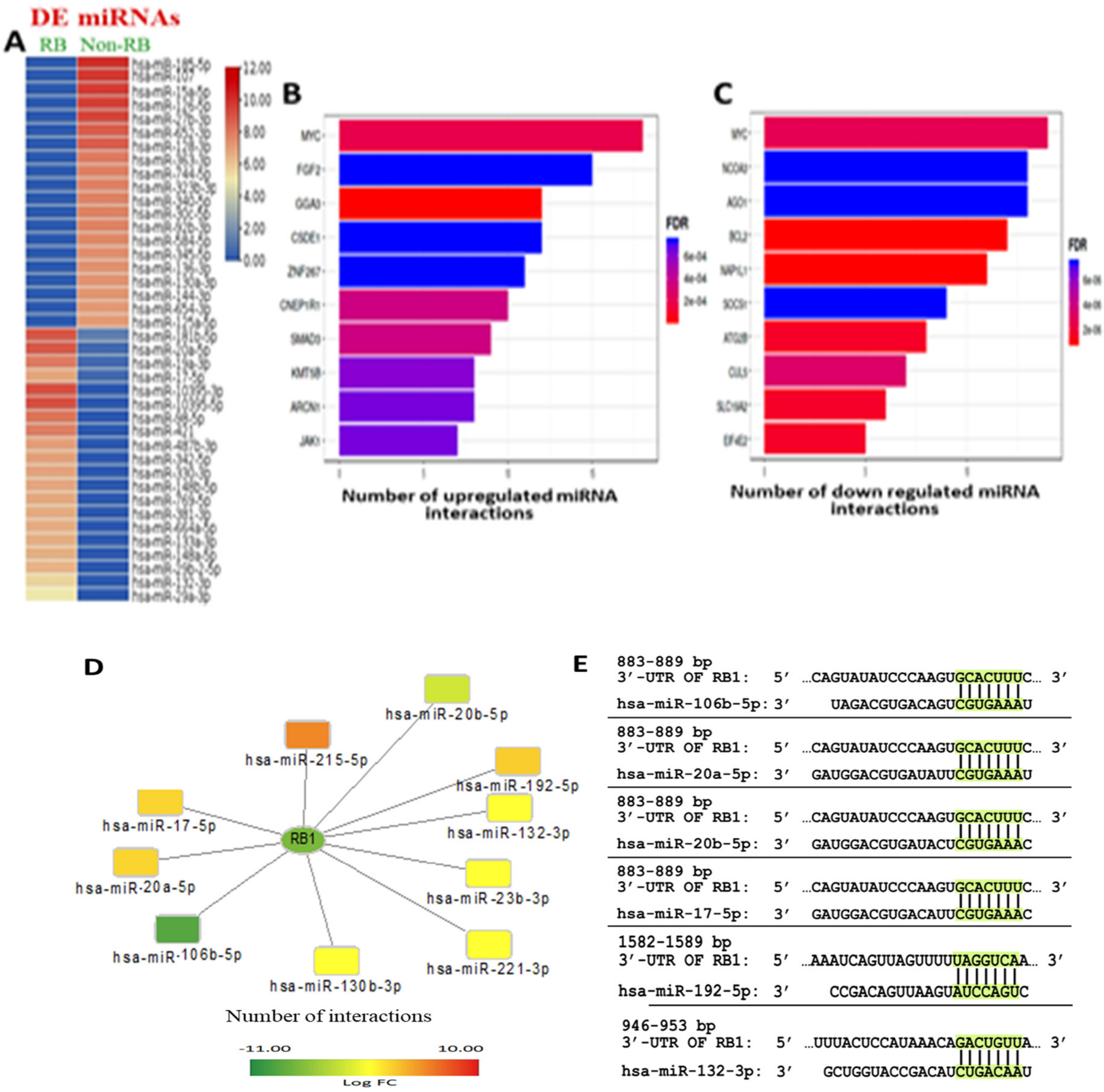

Figure S3. A. Heat map representing the differential expression of miRNAs in RB serum exosomes (sExos) vs non-RB. **B & C.** miRNA-target interactions in RB sExos for up regulated miRNAs and down regulated miRNAs. **D.** Cytoscape network depicting expression profiles of RB1 and its direct miRNA targets in RB sExos. **E.** Schematic illustration of the potential miRNA binding sites in 3′-UTRs of target RB1.

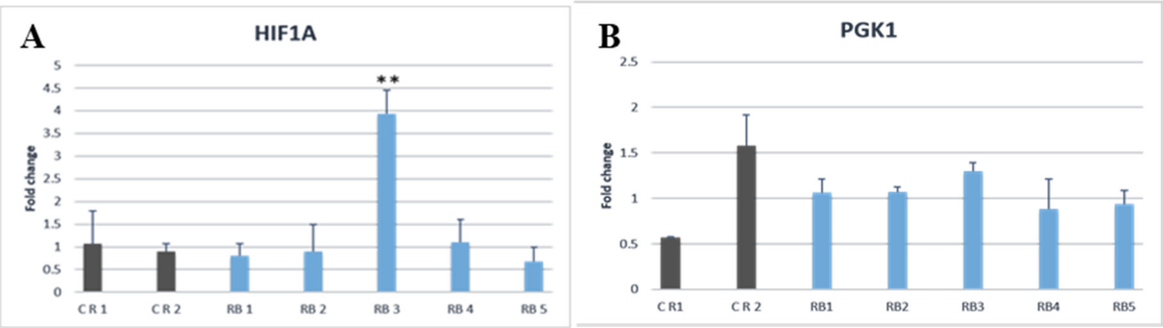

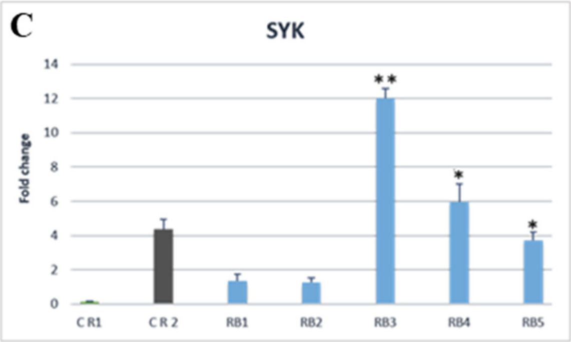

D

Gene	Fold change	FDR
HIF1A	2.1	0.02
PGK1	4.8	0.0001
SYK	1.4	0.1

Figure S4. Real time expression data of A. HIF1A, B. PGK1, and C. SYK in primary retinoblastoma (RB) tumors and control retina (CR). *=p-value<0.05 and **=p-value<0.02 calculated by paired t test. E. RB vs non-RB serum exosomal gene expression by RNA seq.
