# Supplementary material for: Comprehensive Analysis of Serum Small Extracellular Vesicles-Derived Coding and Non-Coding RNAs from Retinoblastoma Patients for Identifying Regulatory Interactions
Source: Cancers (Basel). 2022 Aug 29;14(17):4179. doi: 10.3390/cancers14174179 (PMC9454787; doi:10.3390/cancers14174179)
Supplement: Supplementary file 1 [file cancers-14-04179-s001.zip › cancers-1828690 - Supplementary Tables.pdf]

Table S1: Primers for RT-qPCR

Primer	Forward	Reverse
β-actin	TCACCCACACTGTGCCCATCTACG	CAGCGGAACCGCTCATTGCCAATG
SYK	GGAAGTGAAGTCACCGCTATG	GGGAGCGGTTAGTTCACCAC
HIF1A	CATAAAGTCTGCAACATGGAAGGT	ATTTGATGGGTGAGGAATGGGTT
PGK1	AACCAGAGGATTAAGGCTGC	GCCTACACAGTCCTTCAAGA

Table S2: Clinical characteristics of subjects included in the study

Sample ID	Age	Gender	Laterality	Tumor classification	Cellular differentiation	Health condition	HRF/ No HRF	Choroid invasion	Optic nerve involvement
C1*	5	M	NA	NA	NA	Healthy (Right eye injury by fall from cycle)	NA	NA	NA
C2*	5	M	NA	NA	NA	Healthy (Intermittent exotropia)	NA	NA	NA
C3*	4	F	NA	NA	NA	Healthy (exotropia)	NA	NA	NA
C4	8	F	NA	NA	NA	Healthy (exotropia)	NA	NA	NA
C5	5	M	NA	NA	NA	Healthy (Intermittent exotropia)	NA	NA	NA
RB1	9	M	Unilateral (OS)	Group E	Poor	RB	HRF	Massive	Yes
RB2	1	F	Unilateral (OD)	Group E	NA	RB	No HRF	NA	NA
RB3	2	M	Unilateral (OS)	Group E	Moderately differentiated	RB	HRF	Massive	Yes
RB4*	5	M	Unilateral (OD)	Group D	NA	RB	NA	NA	NA
RB5*	2	M	Unilateral (OS)	Group E	-	RB	HRF	Focal	Yes
RB6*	4	M	Unilateral (OS)	Group E	Undifferentiated RB with mixed pattern	RB	HRF	Massive	No
RB7	4	F	Unilateral (OS)	Group E	Poor	RB	HRF	Massive	No
RB8	1	M	Unilateral (OS)	Group D	NA	RB	NA	NA	No

RB9	4	F	Unilateral (OS)	Group E	Poor	RB	HRF	Massive	No
Control Retina	72	M				Natural death			
Control Retina	86	M				Natural death			

* RNA sequencing was performed on serum derived exosomes from these subjects. Blood was collected prior to the treatment (At the time of diagnosis)

Table S3: The concentration, size distribution and zeta potential of serum exosomes from retinoblastoma (RB) and non-RB (control) subjects

Sample ID	Concentration (particles/ml)	Diameter (nm)	Zeta potential (-mv)
**C1**	6.2×10^{11}	123.6	11.1
**C2**	4.2×10^{11}	140.3	11.6
**C3**	4.8×10^{11}	136.3	10.6
**C4**	5.5×10^{11}	142.3	10.8
**C5**	5.9×10^{11}	132.3	11.1
**RB1**	4.3×10^{11}	136.4	12.1
**RB2**	3.8×10^{11}	107.0	10.7
**RB3**	5.1×10^{11}	128.6	12.0
**RB4**	7.3×10^{11}	107.7	13.9
**RB5**	6.2×10^{11}	113.5	10.8
**RB6**	5.9×10^{11}	123.6	11.5
**RB7**	4.8×10^{11}	142.7	14.2
**RB8**	3.8×10^{11}	121.5	13. 5
**RB9**	8.0×10^{11}	113.8	15.8

Statistical Analysis

	Concentration (Mean)	SD	Std. Error	Size (Mean)	SD	Std. Error	Zeta potential (Mean)	SD	Std. Error
**Non-RB**	5.3×10^{11}	0.8×10^{11}	0.4×10^{11}	135.0	7.4	3.3	-11.0	0.4	0.2
**RB**	5.5×10^{11}	1.5×10^{11}	0.5×10^{11}	121.6	12.5	4.2	-12.7	1.7	0.6
**p-value**		0.82			0.03**			0.02**	

** Significance level: p-value < 0.05 calculated by Welch's T-test

Table S4: RNA-seq details of serum exosomes from retinoblastoma (RB) and non-RB subjects

Sample details	Total Sequences (millions) read count	Filtered Sequences (millions)	Total Bases (Gb)	Total bases after filtering (Gb)	Q20 (%)	Q30 (%)	Average % GC Content	Adapter trimmed (%)	Filtered reads (%)
**Long RNA sequencing details**									
RB	35.3	34.3	5.7	4.8	96.69	90.36	47.2	37.8	97.2
Non-RB	40.1	38.2	6.4	5.3	96.98	92.25	49.2	42.3	95.2
**Small RNA sequencing details**									
RB	109.0	35.0	5.6	1.5	99.1	96.6	51.9	48.4	32.1
Non-RB	76.9	17.4	3.9	747.1	98.9	96.4	51.9	47.6	22.7

Table S5: Top 20 up regulated mRNAs in retinoblastoma serum exosomes

S No	Gene	Function	Log2 fold change	FDR
1	ATP1B1 (ATPase Na+/K+ Transporting Subunit Beta 1)	Ion channel transport and Cardiac conduction	9.7	1.78E-10
2	ANKRD7 (Ankyrin repeat domain-containing protein 7)	Protein binding	9.4	1.28E-9
3	TRIM23 (Tripartite Motif Containing 23)	E3 ubiquitin-protein ligase	9.2	2.9E-09
4	PRR14 (Proline Rich 14)	Muscle organ development	9.1	7.4E-09
5	DICER1 (Ribonuclease III)	Short dsRNA-mediated post-transcriptional gene silencing	9.1	1.1E-08
6	ITGAM (Integrin Subunit Alpha M)	Microglial cell activation, Immune system process	9.0	1.3E-08
7	CEPT1(Choline/Ethanolamine Phospho transferase 1)	Phosphatidylcholine and phosphatidyl ethanolamine biosynthesis	9.0	1.5E-08
8	SPATA6 (Spermatogenesis Associated 6)	Involved_in spermatogenesis	9.0	1.8E-08
9	ZNF133 (Zinc Finger Protein 133)	DNA-binding transcription factor activity	8.9	2.5 E-08
10	DLGAP4 (DLG Associated Protein 4)	Molecular organization of synapses and neuronal cell signaling	8.9	4.1E-08
11	LPCAT2 (Lysophosphatidylcholine Acyltransferase 2)	Acyltransferase activity	8.9	4.1E-08
12	FASTKD3 (Fas-activated serine/threonine kinase domain)	For normal mitochondrial respiration	8.9	4.1E-08
13	HIRIP3 (HIRA interacting protein 3)	Chromatin assembly or disassembly	8.9	4.1E-08
14	ATP6V1B1 (ATPase H+ Transporting V1 Subunit B1)	Ion channel transport	8.8	4.8E-08

15	LYG1 (Lysozyme G1)	Metabolic process	8.8	5.7E-08
16	BTG2 (BTG Anti-Proliferation Factor 2)	Regulation of G1/S transition of the cell cycle	8.8	5.7E-08
17	FOLR2 (Folate Receptor Beta)	Folate metabolism, positive regulation of cell population proliferation	8.8	5.7E-08
18	NIMIK (NIM1 Serine/Threonine Protein Kinase)	Serine/Threonine Protein Kinase activity	8.8	6.5E-08
19	CAMKMT (Calmodulin-Lysine N-Methyltransferase)	Methyltransferase activity	8.8	8.0E-08
20	PSAP (Prosaposin)	Lipid metabolic process	8.8	8.0E-08

Table S6: Top 20 down regulated mRNAs in retinoblastoma serum exosomes

S No	Gene name	Function	Log2 fold change	FDR
1	TTC19 (Tetratricopeptide Repeat Domain 19)	Maintain functional integrity of mitochondrial respiratory complex III	-8.83	4.8E-08
2	TRIOBP (TRIO And F-Actin Binding Protein)	Ubiquitin protein ligase binding	-8.8	6.5E-08
3	LIG3 (DNA Ligase 3)	DNA strand-break repair, mitochondrial base-excision DNA repair	-8.7	9.6E-08
4	DOT1L (DOT1 Like Histone Lysine Methyltransferase)	transcription factor binding, histone methyltransferase activity	-8.5	3.2E-07
5	UPF1 (RNA Helicase And ATPase)	mRNA nuclear export and mRNA surveillance	-8.5	4.8E-07
6	RPS6KA2 (Ribosomal Protein S6 Kinase A2)- serine/threonine kinases	Acts downstream of ERK (MAPK1/ERK2 and MAPK3/ERK1) signaling Tumor suppressor in epithelial ovarian cancer cells	-8.5	4.8E-07
7	PPP1R9 Protein Phosphatase 1 Regulatory Subunit 9B	Putative tumor suppressor function, ERK signaling	-8.4	7.6E-07
8	CEP76 (Centrosomal Protein 76)	Regulation of G2/M transition of mitotic cell cycle	-8.4	9.6E-07
9	SMPD3 (Sphingomyelin Phosphodiesterase 3)	Signaling by GPCR and IL-2 Pathway	-8.4	9.6E-07
10	PPP2R5B Protein Phosphatase 2 Regulatory Subunit B'Beta	RET and ERK signaling, Ser/Thr phosphatases	-8.3	1.8E-06
11	MAP3K20 Mitogen-Activated Protein Kinase 20	MAPKKK family of signal transduction , regulation of S and G2 cell cycle checkpoint by direct phosphorylation of CHEK2	-8.3	1.8E-06
12	AKAP8 (A-Kinase Anchoring Protein 8)	Cell cycle Chromosome condensation in prometaphase and Activation of cAMP-	-8.1	1.2E-07

		Dependent PKA		
13	PPP2R5C Protein Phosphatase 2 Regulatory Subunit B'Gamma Ser/Thr phosphatases	Implicated in the negative control of cell growth and division	-8.0	1.7E-07
14	SEH1L (SEH1 Like Nucleoporin), Nup107-160 Subcomplex Subunit SEH1	Required for normal kinetochore microtubule attachment, mitotic progression and chromosome segregation	-7.9	3.5E-07
15	TSG101 Tumor Susceptibility 101, Component of the ESCRT-I complex	Regulator of vesicular trafficking process	-7.9	5.0E-07
16	CCNJ (Cyclin J)	Cyclin-dependent protein serine/threonine kinase regulator activity	-7.8	3.1E-05
17	RBBP8 (RB Binding Protein 8, Endonuclease)	RB binding protein, Transcriptional regulation, DNA repair, and/or cell cycle checkpoint control	-7.8	4.3E-05
18	CDC6 (Cell Division Cycle 6)	E2F mediated regulation of DNA replication	-7.7	2.4E-06
19	PPP5C (Protein Phosphatase 5 Catalytic Subunit)	Regulation of cell growth and differentiation	-7.6	3.6E-06
20	TRIP13(Thyroid Hormone Receptor Interactor 13)	Transcription co-regulator activity	-7.6	3.6E-06

Table S7: Top 20 upregulated miRNAs in retinoblastoma serum exosomes

	Gene name	Log2 FC	FDR	miR family	Chromosome location	Number of target genes dysregulated in RB serum exosomes
1	hsa-miR-10395-3p	12.4	9.32E-29	-	chr19: 12703601-12703664 [-]	-
2	hsa-miR-10395-5p	12.4	9.32E-29	-	chr19: 12703601-12703664 [-]	-
3	hsa-miR-98-5p*	11.4	2.34E-22	let-7	chrX: 53557192-53557274 [-]	574
4	hsa-miR-421	11.2	1.31E-20	mir-95	chrX: 74218377-74218461 [-]	182
5	hsa-miR-487b-3p	10.2	5.56E-15	mir-154	chr14: 101046455-101046538 [+]	17
6	hsa-miR-342-5p	10.2	9.82E-15	mir-342	chr14: 100109655-100109753 [+]	89
7	hsa-miR-330-3p	10.1	2.13E-14	mir-330	chr19: 45638994-45639087 [-]	104
8	hsa-miR-148b-5p	10.1	5.64E-14	mir-148	chr12: 54337216-54337314 [+]	40
9	hsa-miR-769-5p	10.0	1.17E-13	mir-769	chr19: 46018932-46019049 [+]	149
10	hsa-miR-381-3p	9.9	2.03E-13	mir-154	chr14: 101045920-101045994 [+]	98
11	hsa-miR-664a-5p	9.9	2.79E-13	mir-664	chr1: 220200538-220200619 [-]	90
12	hsa-miR-133a-3p	9.8	5.71E-13	mir-133	chr18: 21825698-21825785 [-]	85
13	hsa-miR-148a-5p	9.8	1.26E-12	-	-	62
14	hsa-miR-29b-2-5p	9.7	5.30E-12	mir-29	chr1: 207802443-207802523 [-]	88
15	hsa-miR-20a-5p*	9.5	2.19E-27	miR-17	chr13: 91351065-91351135 [+]	959
16	hsa-miR-132-3p	8.8	4.63E-08	mir-132	chr17: 2049908-2050008 [-]	174
17	hsa-miR-19a-3p*	8.6	3.76E-21	mir-19	chr13: 91350891-91350972 [+]	561
18	hsa-miR-29a-3p	8.3	6.66E-06	mir-29	chr7: 130876747-130876810 [-]	178

19	hsa-miR-181b-5p	8.2	1.27E-27	mir-181	chr1: 198858873-198858982 [-]	343
20	hsa-miR-17-5p*	7.5	4.13E-14	miR-17	chr13: 91350605-91350688 [+]	1022

*miRNAs that are expressed in retinoblastoma tumor tissues

Table S8: Top 20 down regulated miRNAs in retinoblastoma serum exosomes

S. No	Gene name	Log2 (fold change)	FDR	miR family	Chromosome location	Number of gene interactions
1	hsa-miR-185-5p	-13.3	4.92E-30	mir-185	chr22: 20033139-20033220 [+]	269
2	hsa-miR-107	-13.0	1.38E-28	mir-103	chr10: 89592747-89592827 [-]	281
3	hsa-miR-15a-5p	-12.9	4.97E-28	mir-15	chr13: 50049119-50049201 [-]	674
4	hsa-miR-126-5p	-12.8	2.46E-27	mir-126	chr9: 136670602-136670686 [+]	102
5	hsa-miR-27b-3p	-12.6	2.16E-26	mir-27	chr9: 95085445-95085541 [+]	359
6	hsa-miR-652-3p	-12.1	2.34E-22	mir-652	chrX: 110055329-110055426 [+]	133
7	hsa-miR-128-3p	-12.0	4.39E-22	mir-128	chr2: 135665397-135665478 [+]	434
8	hsa-miR-363-3p	-11.2	5.86E-18	mir-363	chrX: 134169378-134169452 [-]	337
9	hsa-miR-744-5p	-11.1	4.60E-17	mir-744	chr17: 12081899-12081996 [+]	317
10	hsa-miR-323b-3p	-11.1	7.58E-17	mir-154	chr14: 101056219-101056300 [+]	34
11	hsa-miR-340-5p	-11.0	8.70E-17	mir-340	chr5: 180015303-180015397 [-]	304
12	hsa-miR-30c-5p	-11.0	1.00E-16	mir-30	chr6: 71376960-71377031 [-]	402
13	hsa-miR-92b-3p	-10.9	1.70E-16	mir-25	chr1: 155195177-155195272 [+]	638
14	hsa-miR-584-5p	-10.8	7.98E-16	mir-584	chr5: 149062313-149062409 [-]	60
15	hsa-miR-345-5p	-10.7	6.76E-15	mir-345	chr14: 100307859-100307956 [+]	31
16	hsa-miR-136-3p	-10.6	1.92E-14	mir-136	chr14: 100884702-100884783 [+]	40
17	hsa-miR-130a-3p	-10.6	2.80E-14	mir-130	chr11: 57641198-57641286 [+]	392
18	hsa-miR-144-3p	-10.5	5.48E-14	mir-144	chr17: 28861533-28861618 [-]	187
19	hsa-miR-654-3p	-10.4	2.26E-13	mir-654	chr14: 101040219-101040299 [+]	51
20	hsa-miR-125a-5p	-10.4	3.56E-13	mir-10	chr19: 51693254-51693339 [+]	220

Table S9: Dysregulated microRNAs identified in RB serum exosomes and their expression in RB tumor tissues from review of literature

S No	miRNA	Zhao et al 2009 (RB vs retina) [26]	Conkrite et al 2011 (RB vs FW18 retina) [27]	Huang et al 2007 (RB vs retina) [28]	Beta et al 2019 Insilico analysis RB vs control serum [29]	Present study RB vs non-RB serum exosomes
1	hsa-miR17-5p		neutral	up		up
2	hsa-miR-20a			up		20a-5p up
3	miR-106b		neutral		up	106b-5p-down<br>106b-3p-neutral
4	miR-93				up	93-5p -neutral
5	miR-16		up		up	16-5p-up
6	hsa-let-7a	neutral		down		7a-3p -down

						7a-5p -up
7	let-7b			down		7b-5p- neutral
8	hsa-let-7c	neutral		down		7c-5p-up
9	hsa-let-7d				down	7d-3p-down<br>7d-5p-neutral
10	hsa-let-7e				down	7e-5p -down
11	hsa-let-7f		down		down	7f-5p-up
12	miR-25		up	up		25-3p-down
13	miR-142-5p					up
14	miR-324-5p	neutral				up
15	miR-29a	down				29a-3p-up
16	miR-182		up	down		182-5p-up
17	hsa-miR-101					
18	miR-148b		up			148b-5p-up<br>148b-3p-down
19	miR-143		up			143-3p down
20	miR-19a				Up (RL)	19a-3p-up
21	miR-19b				neutral	19b-3p-up
22	miR-181a			down		181a-5p- up<br>181a-3p-down
23	miR-221				down	221-3p-down

Note: Previous papers not specified the arm specific miRNAs expression (5p-arm or 3p-arm). Thus, to avoid ambiguity we have listed expression profiles of both their expression.

Table S10: Dysregulated lncRNAs identified in retinoblastoma (RB) serum exosomes and their expression in RB tumor tissues from review of literature

S No	lncRNA	Up/down regulated	RB tissues/cell lines	Ref	Expression status in RB vs non-RB exos (Fold change)
1	AFAP1-AS1	Up	RB tissue	[30]	Up (5.94)
2	BANCR	Up	RB tissue	[31]	Up (4.0)
3	BDNF-AS	down	RB tissue	[32]	Up (2.5)
4	HOTAIR	Up	RB tissues, RB cell lines (HXO-RB44, and Y79)	[33]	Up (1.6)

5	MALAT1	Up	RB tissues, RB cell lines (ARPE-19, HXO-RB44, WERI-RB-1, SO-RB50, and Y79)	[34]	Down (-6.9)
6	MEG3	Down	RB tumor tissues	[35]	Neutral (1.7)
7	NEAT1	Up	RB tissues, RB cell lines (Y79, Weri-Rb1, and SO-RB50)	[36]	Neutral (-1.4)
8	XIST	Up	RB tissues	[37]	Neutral (1.0)
9	ELFN1-AS1	Up	RB tissues and RB cell lines	[38]	Down (-5.9)
10	PANDAR	Up	RB tissues and WERI-Rb1, Y79, SO-Rb50 and HXO-RB44 cells	[39]	Neutral (-4.9)
11	UCA1	Up	Carboplatin resistant RB cells developed from WERI-RB-1 and Y79 cells	[40]	Neural (-3.9)
12	DANCR	Up	RB tissues and cell lines	[41]	Neutral (0.1)
13	FEZF1-AS1	Up	Y79	[42]	UP (2.4)
14	SNHG-16	Up	RB tissues and WERI-RB1, SO-RB50, Y79	[43]	Down (-2.3)
